# Gyri of the human parietal lobe: Volumes, spatial extents, automatic labelling, and probabilistic atlases

**DOI:** 10.1371/journal.pone.0180866

**Published:** 2017-08-28

**Authors:** Heather M. Wild, Rolf A. Heckemann, Colin Studholme, Alexander Hammers

**Affiliations:** 1 Neurodis Foundation, Lyon, France; 2 Univ Lyon, Université Claude Bernard Lyon 1, Inserm, Stem Cell and Brain Research Institute U1208, Bron, France; 3 MedTech West at Sahlgrenska University Hospital, University of Gothenburg, Gothenburg, Sweden; 4 Department of Pediatrics, Division of Neonatology, University of Washington, Seattle, Washington, United States of America; 5 Division of Imaging Sciences and Biomedical Engineering, King’s College London, London, United Kingdom; University of Cambridge, UNITED KINGDOM

## Abstract

Accurately describing the anatomy of individual brains enables interlaboratory communication of functional and developmental studies and is crucial for possible surgical interventions. The human parietal lobe participates in multimodal sensory integration including language processing and also contains the primary somatosensory area. We describe detailed protocols to subdivide the parietal lobe, analyze morphological and volumetric characteristics, and create probabilistic atlases in MNI152 stereotaxic space. The parietal lobe was manually delineated on 3D T1 MR images of 30 healthy subjects and divided into four regions: supramarginal gyrus (SMG), angular gyrus (AG), superior parietal lobe (supPL) and postcentral gyrus (postCG). There was the expected correlation of male gender with larger brain and intracranial volume. We examined a wide range of anatomical features of the gyri and the sulci separating them. At least a rudimentary primary intermediate sulcus of Jensen (PISJ) separating SMG and AG was identified in nearly all (59/60) hemispheres. Presence of additional gyri in SMG and AG was related to sulcal features and volumetric characteristics. The parietal lobe was slightly (2%) larger on the left, driven by leftward asymmetries of the postCG and SMG. Intersubject variability was highest for SMG and AG, and lowest for postCG. Overall the morphological characteristics tended to be symmetrical, and volumes also tended to covary between hemispheres. This may reflect developmental as well as maturation factors. To assess the accuracy with which the labels can be used to segment newly acquired (unlabelled) T1-weighted brain images, we applied multi-atlas label propagation software (MAPER) in a leave-one-out experiment and compared the resulting automatic labels with the manually prepared ones. The results showed strong agreement (mean Jaccard index 0.69, corresponding to a mean Dice index of 0.82, average mean volume error of 0.6%). Stereotaxic probabilistic atlases of each subregion were obtained. They illustrate the physiological brain torque, with structures in the right hemisphere positioned more anteriorly than in the left, and right/left positional differences of up to 10 mm. They also allow an assessment of sulcal variability, e.g. low variability for parietooccipital fissure and cingulate sulcus. Illustrated protocols, individual label sets, probabilistic atlases, and a maximum-probability atlas which takes into account surrounding structures are available for free download under academic licences.

## Introduction

Seminal neuroanatomical studies have shown the complexity of the human cerebral cortex based on single example brains [1,2], as well as variability on dissection of 25 brains, documented on photographs and published in book form [3]. 3D digital brain atlases have been produced [4–14], and more recently still, single subject atlases have been combined into multi-subject, maximum probability and/or probabilistic atlases [15–19].

The parietal lobe contains the primary somatosensory cortex but also multimodal regions, receiving information from somesthetic, auditory, and visual neocortices. Its role in language processing is well established [20,21]. It also plays a role in mathematical cognition [22–24], and is possibly involved in early-stage romantic love [25], schizophrenia [26], thought disorders [27], and creativity [28]. The junction of the parietal and temporal lobes has been implicated in complex social cognition [29,30].

**Fig 1:**
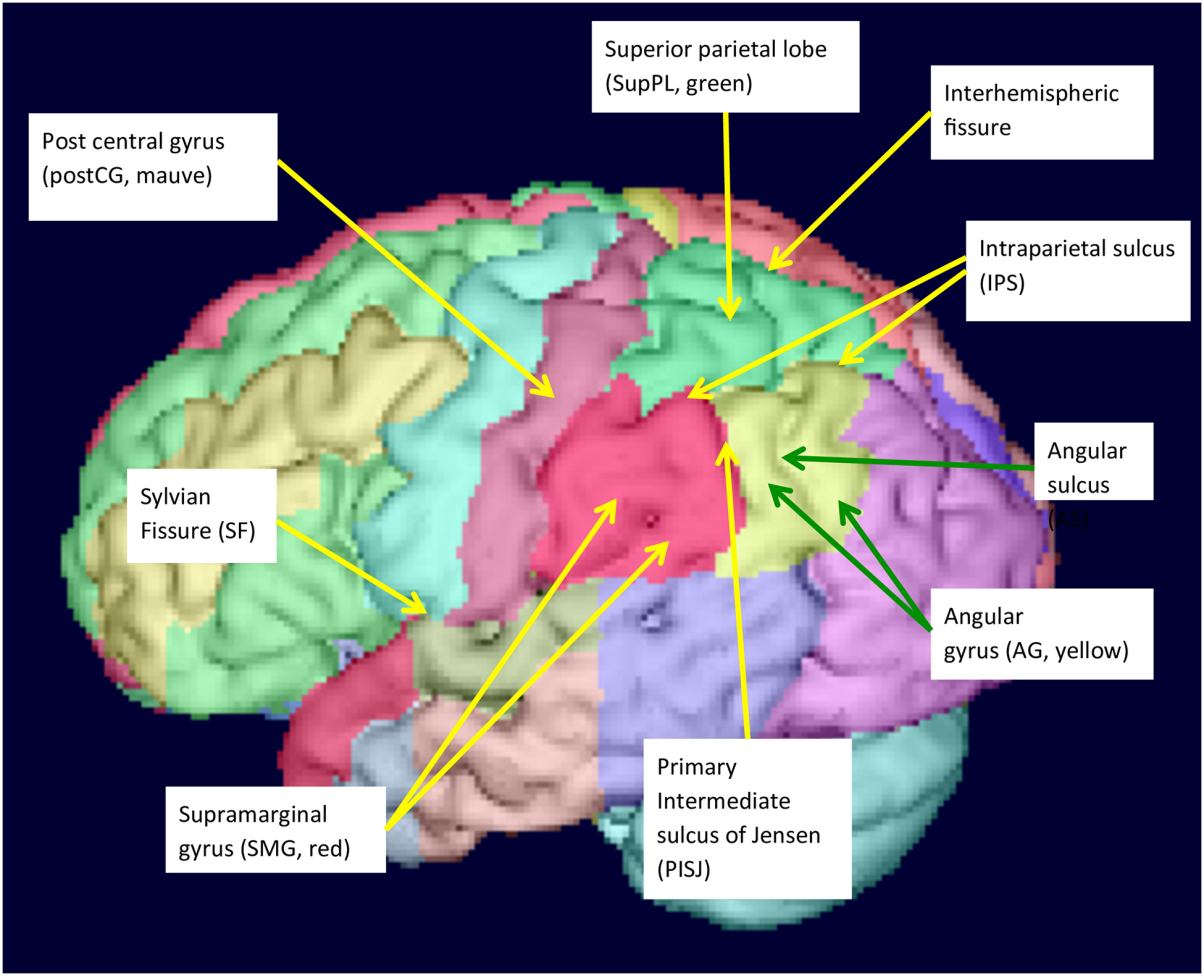
Surface view of parietal lobe structures.

Globally, the inferior parietal cortex (IPC) is separated from the superior parietal cortex (SPC) by the intraparietal sulcus (IPS) (Fig 1).

The present study subdivides the parietal cortex into four structures, namely, the supramarginal gyrus (SMG), angular gyrus (AG), superior parietal lobe (supPL) and postcentral gyrus (postCG). The choice of regions was based on traditional description of parietal neuroanatomy, presence of consistent sulcal landmarks [3] that allow consistent segmentation, combined with progress in visualisation and segmentation software that now allows simultaneous viewing of tomographic and surface images.

Both the SMG and AG are involved in language processing, the SMG more in phonological and the AG more in semantic aspects [31–39]. To separate SMG and AG, most neuroanatomical studies use the primary intermediate sulcus of Jensen (PISJ), which branches from the IPS (LONI resource research protocols; [40–43]). Some studies point out the highly variable nature of the PISJ [44,45], however, they also suggest that when the PISJ is present, it may indicate a cytoarchitectonic boundary. The superior border of both the SMG and the AG is formed by the IPS. The SMG forms a horseshoe shape embracing the superior end of the Sylvian Fissure (SF). The AG, which lies posterior to the SMG, similarly forms a horseshoe shape around the ascending branch of the superior temporal sulcus (STS). This branch is known as the angular sulcus (AS). Although the path of the STS is often complicated, certain patterns have been described [46]. The anterior border of the SMG is the postcentral sulcus (postCS). The AG ends posteriorly at the occipital lobe (OL).

In this study, we developed delineation protocols and analyzed morphology and morphometry of the parietal lobe neuroanatomical subdivisions based on 30 MRIs. From the resulting segmentations, we produced probabilistic maps for future functional studies. We also determined the accuracy of automatic segmentations when the 30 individual manual parietal lobe segmentations are used as input for an automatic multi-atlas labelling technique.

## Materials and methods

### Data sets

The data sets [16,47] are from healthy volunteers from the database at the National Society for Epilepsy’s MRI Unit. They had no neurological, medical, or psychiatric condition and their MRIs had been read as normal by two experienced neuroradiologists. Ethical approval had been obtained from the Joint Medical Ethics Committee of the Institute of Neurology and the National Hospital for Neurology and Neurosurgery, University College London, Queen Square, and all subjects had given written informed consent.

Data sets consisted of 30 MRIs from healthy volunteers (15 women), scanned using a 1.5 Tesla GE Signa Echospeed scanner (GE Healthcare, Little Chalfont, Buckinghamshire, UK). The inversion recovery prepared fast spoiled gradient recall T1-weighted sequence yielded images with voxel sizes 0.9375 × 0.9375 × 1.5 mm^3^, resliced in AC-PC orientation using windowed sinc interpolation to isotropic voxels with 0.9375 mm edge length. The median age was 30 years (range 20–54); individual demographic characteristics of the sample are given in Table 1.

**Table 1:** Subject and brain characteristics.

ID	ICV	Brain	Age	Gender
	cm^3^	cm^3^	years	1 = female
a01	1250	1055	36	1
a02	1414	1177	26	0
a03	1386	1159	53	0
a04	1558	1309	38	0
a05	1629	1454	25	0
a06	1458	1218	33	0
a07	1284	1095	32	1
a08	1468	1281	23	0
a09	1325	1148	24	1
a10	1436	1286	20	0
a11	1428	1193	38	1
a12	1634	1401	36	0
a13	1424	1260	29	1
a14	1302	1093	54	1
a15	1269	1088	23	1
a16	1746	1474	29	0
a17	1379	1206	20	1
a18	1273	1141	26	1
a19	1533	1339	34	1
a20	1869	1569	32	0
a21	1458	1286	30	0
a22	1412	1222	29	1
a23	1531	1328	30	0
a24	1321	1153	32	1
a25	1351	1136	31	1
a26	1582	1356	33	0
a27	1400	1206	31	1
a28	1653	1358	30	0
a29	1693	1460	23	0
a30	1528	1306	31	1
**Mean**	1466	1259	31	
**SD**	153.3	129.4	7.8	
**CV%**	10	10	25	
**Sum%**				50

IPS IPS discontinuous = 0, continuous = 1 IPS/PCG IPS to PCG, disconnected = 0, connected = 1 GMWM Combined grey and white matter volume in mm^3^ GM Grey matter volume in mm^3^ SF or AS Sylvian fissure or Angular sulcus: short = 0 (i.e. sulcus <50% of the vertical height of the parietal lobe), long = 1 (sulcus >50% of the vertical height). Sup Superior limit of region (slice number in RView) Inf Inferior limit of region (slice number in RView) Top Top of SF or AS Pct Percentage height of SF or AS *G_ant Number of additional gyri anteriorly *G-sup Number of additional gyri superiorly *G_post Number of additional gyri posteriorly *G_Total Total number of additional gyri PISJ Prominence of PISJ: absent = 0, dimple = 1, vague = 2, prominent = 3 AS/IPS AS to IPS: unconnected = 0, connected = 1 SMG Supramarginal gyrus AG Angular gyrus PostCG Postcentral gyrus SupPL Superior parietal lobe L Left hemisphere R Right hemisphere SD Standard deviation CV% Coefficient of variation (SD/mean), expressed as a percentage Sum% Percentage of subjects having a score of 1

The series of MRI scans and manual delineations has previously been used to produce maximum probability atlases of the human brain [16,19]; to automatically label any T1-weighted brain image [48–50]; and to produce probabilistic atlases of the thalamus, basal ganglia, and inferior frontal gyrus [15,47].

### Delineation software

We used RView software [51] to delineate regions, construct 3D models, and calculate cortical volumes for each region. The software provides three orthogonal viewing planes, transverse, sagittal, and coronal, in addition to a 3D-rendered surface view that can be tilted at arbitrary angles, permitting optimal viewing of the parietal convexity, which is hard to visualize on traditional orthogonal slices. The surface view provided by the current RView software occasionally indicated that changes were needed where the previously described boundaries had not been sufficiently accurate. Even though the most suitable plane to define the SMG and AG is thought to be the coronal plane [52], each of the orientations was referred to in this work, and in difficult cases a consensus decision was reached through discussion between the main investigator (HMW) and the senior investigator with a >20-year track record of developing protocols and performing delineations (AH).

### Manual segmentation procedure overview

Using the previously published Hammers_mith atlases as a starting point, new delineation protocols were developed for SMG and AG which could be applied to all 60 hemispheres. They are given and illustrated in the supplementary S2 File.

The Hammers_mith atlas (www.brain-development.org) protocol descriptions in Gousias et al. [19] and Hammers et al. [16]) delineate 83 regions.

In the 83-region version, the external boundaries of the parietal cortex had already been defined. The parietal lobe had been segmented into SupPL (regions 62 and 63; odd numbers designating the right hemisphere throughout), post CG (regions 60 and 61), and IPC (regions 32 and 33). Broadly speaking, the postCG borders on the central sulcus anteriorly and the postcentral sulcus (postCS) posteriorly; it is delineated on transverse planes so the white matter part of the region contains most of the afferent somatosensory fibres. The superior parietal lobe includes the medial hemispheric wall excluding the cingulate gyrus; on the lateral convexity the inferior boundary is the intraparietal sulcus. The inferior parietal lobe has no clear anatomically defined inferior boundary with the temporal lobe; an anatomically informed artificial plane was used [16]: All images had been reoriented manually such that anterior and posterior commissure were aligned horizontally. On transverse slices, the artificial plane had been defined as the superior border of the posterior temporal lobe; this in turn was the superiormost slice on which the posterior borders of four previously defined temporal lobe structures (parahippocampal/ambient gyri; superior temporal gyri; middle/inferior temporal gyri; fusiform gyri) occupied >50% of the space between CSF laterally and non-temporal lobe structures medially (p. 243 in [16]).

To further divide the inferior parietal lobe and delineate SMG and AG, we used established cortical landmarks as regional boundaries. The origin of the PISJ marks the superior division between the SMG and AG and was used as the posterior boundary of SMG (see Introduction and SMG protocol in the supplementary S2 File). The PISJ is similarly considered a boundary indicator by other authors [40–42,44]. The subdivision results in the two horseshoe-shaped regions of the SMG and AG enclosing the SF and AS respectively. Although the PISJ was not prominent in many brains, it was at least vaguely discernible or seen clearly forming a dimple in all but one hemisphere.

As in our previous work, protocols incorporated grey and white matter portions and were first established based on literature review and test cases; then the protocols were applied to all 60 hemispheres, one structure at a time; and finally structures were reviewed for protocol adherence. In total, the process took approximately six months.

### Morphological and volumetric evaluation

Several morphological characteristics were noted (see Tables 1–3).

**Table 2:** Morphological characteristics of left parietal lobe and volumes of left supramarginal and angular gyri.

			L SMG												L AG											
ID	IPS	IPS/PCG	GMWM	GM	SF	sup	inf	top	pct	*G ant	*G sup	*G post	*G Total	PISJ	GMWM	GM	AS	sup	inf	top	pct	*G ant	*G sup	*G post	*G Total	AS/IPS
a01	0	1	39157	18225	1	42	82	57	63	1	0	2	3	3	32437	19572	1	38	82	50	73	1	1	1	3	0
a02	0	1	42282	20162	1	34	82	46	75	1	1	1	3	3	17969	11313	1	43	82	53	74	0	1	1	2	0
a03	1	1	35039	16605	1	43	75	55	63	0	1	1	2	3	17118	9572	1	45	75	59	53	1	0	1	2	0
a04	0	0	44817	20723	0	42	84	68	38	2	2	0	4	3	17098	10689	1	45	84	49	90	0	0	0	0	0
a05	0	1	44688	21519	1	40	82	57	60	1	2	1	4	2	10986	6993	1	44	82	61	55	0	2	0	2	0
a06	1	1	35330	17709	1	42	85	61	56	1	1	0	2	1	19946	12585	1	45	85	52	77	1	0	1	2	0
a07	1	1	29373	15083	1	45	79	61	53	0	0	1	1	3	33973	18512	1	43	79	49	83	0	1	1	2	0
a08	1	1	37233	18839	1	43	80	59	57	1	1	1	3	3	12872	8255	1	46	80	61	56	2	1	0	3	0
a09	0	1	35373	18637	1	42	85	58	63	1	1	1	3	2	21337	13480	1	38	85	52	70	1	1	1	3	1
a10	1	1	36541	18920	0	43	80	66	38	2	2	0	4	1	17290	11671	1	43	80	55	68	2	1	0	3	0
a11	0	1	25515	13321	1	51	86	63	66	0	1	1	2	3	15086	9423	1	57	86	63	79	2	0	0	2	1
a12	1	1	32994	16382	1	40	78	56	58	0	2	1	3	3	19281	11978	1	43	78	47	89	0	0	2	2	0
a13	1	1	33122	17020	1	42	77	46	89	1	1	0	2	2	13109	8424	1	45	77	51	81	1	0	0	1	0
a14	1	1	29697	14249	0	46	78	63	47	1	1	0	2	3	17165	10456	1	48	78	56	73	2	0	0	2	0
a15	0	0	28610	16234	0	42	73	59	45	1	1	0	2	3	18218	11806	1	41	73	43	94	0	0	0	0	1
a16	1	1	33494	18404	1	42	83	56	66	2	2	0	4	1	28374	17192	1	44	83	59	62	3	0	0	3	0
a17	0	1	29366	15255	1	39	73	43	88	1	0	1	2	2	30108	16963	1	34	73	42	79	1	1	2	4	0
a18	0	0	42690	22667	0	35	80	63	38	2	1	0	3	2	20422	13389	1	41	80	48	82	0	0	1	1	0
a19	1	1	35080	18404	1	59	91	63	88	2	0	1	3	3	20331	13024	1	52	91	61	77	1	1	1	3	0
a20	0	0	43247	21644	1	40	83	51	74	3	0	0	3	3	22083	12292	1	35	83	45	79	0	1	0	1	1
a21	0	1	38212	19528	1	42	81	59	56	0	2	0	2	3	20422	12893	1	40	81	53	68	0	1	0	1	0
a22	0	1	41205	20189	0	42	85	73	28	1	2	0	3	3	26163	16325	1	50	85	56	83	0	0	2	2	0
a23	1	1	32805	16804	1	48	89	62	66	0	2	1	3	2	28623	16521	1	45	89	55	77	2	0	1	3	0
a24	1	1	42201	20267	1	44	83	54	74	0	1	2	3	3	21360	13129	1	50	83	59	73	1	0	0	1	0
a25	1	1	33888	17000	1	46	81	51	86	2	0	0	2	3	30837	19389	1	42	81	56	64	0	2	1	3	1
a26	1	1	34925	16963	1	51	90	54	92	0	0	0	0	3	29508	16939	1	49	90	52	93	0	0	4	4	0
a27	0	1	36389	18043	0	45	80	65	43	1	2	1	4	2	13740	8539	1	49	80	55	81	0	0	1	1	0
a28	0	1	35728	17250	0	55	89	73	47	0	2	0	2	2	15063	9781	1	58	89	64	81	0	0	2	2	1
a29	1	1	37047	19369	1	44	83	52	79	0	1	2	3	1	18390	11576	1	51	83	54	91	0	0	1	1	0
a30	1	1	39761	19754	0	48	87	69	46	2	2	0	4	2	14847	9295	1	54	87	70	52	0	1	0	1	0
**Mean**			36194	18172		44	82	59	61						20805	12732		45	82	54	75					
**SD**			4985	2221		5.2	4.6	7.4	17.4						6346	3497		5.8	4.6	6.4	11.6					
**CV%**			14	12		12	6	13	28						31	27		13	6	12	15					
**Median**										1.0	1.0	0.5	3.0	3.0								0.0	0.0	1.0	2.0	
**Sum%**	53	87			70												100									20
% bigger than R			9.5	9.3																						

IPS IPS discontinuous = 0, continuous = 1 IPS/PCG IPS to PCG, disconnected = 0, connected = 1 GMWM Combined grey and white matter volume in mm^3^ GM Grey matter volume in mm^3^ SF or AS Sylvian fissure or Angular sulcus: short = 0 (i.e. sulcus <50% of the vertical height of the parietal lobe), long = 1 (sulcus >50% of the vertical height). Sup Superior limit of region (slice number in RView) Inf Inferior limit of region (slice number in RView) Top Top of SF or AS Pct Percentage height of SF or AS *G_ant Number of additional gyri anteriorly *G-sup Number of additional gyri superiorly *G_post Number of additional gyri posteriorly *G_Total Total number of additional gyri PISJ Prominence of PISJ: absent = 0, dimple = 1, vague = 2, prominent = 3 AS/IPS AS to IPS: unconnected = 0, connected = 1 SMG Supramarginal gyrus AG Angular gyrus PostCG Postcentral gyrus SupPL Superior parietal lobe L Left hemisphere R Right hemisphere SD Standard deviation CV% Coefficient of variation (SD/mean), expressed as a percentage Sum% Percentage of subjects having a score of 1

**Table 3:** Morphological characteristics of right parietal lobe and volumes of right supramarginal and angular gyri.

			R	SMG											R	AG										
ID	IPS	IPS/PCG	GMWM	GM	SF	sup	inf	top	pct	*G ant	*G sup	*G post	*G Total	PISJ	GMWM	GM	AS	sup	inf	top	pct	*G ant	*G sup	*G post	*G Total	AS/ IPS
a01	0	1	32559	16629	1	40	72	52	63	1	0	2	3	3	30152	17830	1	33	72	44	72	1	1	2	4	1
a02	0	1	32177	15802	1	43	76	47	88	3	0	1	4	2	20010	12329	1	44	76	56	63	1	1	1	3	0
a03	0	1	31168	14192	1	43	74	53	68	0	0	1	1	3	19302	11246	1	42	74	51	72	1	0	2	3	1
a04	0	0	27739	12464	0	50	82	68	44	1	1	1	3	3	21988	12720	1	42	82	47	88	0	0	3	3	0
a05	0	1	31155	15056	0	40	81	65	39	0	1	0	1	3	23757	13969	1	40	81	54	66	1	1	2	4	0
a06	0	1	25839	12812	1	48	77	57	69	0	1	0	1	1	19653	12261	1	46	77	53	77	0	0	1	1	1
a07	1	1	30777	14961	0	42	80	70	26	0	1	1	2	0	31273	18664	1	44	80	45	97	0	0	0	0	0
a08	1	1	35181	17526	0	40	77	62	41	1	2	1	4	2	12450	8485	1	46	77	46	100	1	0	0	1	1
a09	0	1	33915	17830	1	37	84	47	79	0	1	2	3	3	24381	15697	1	44	84	59	63	1	1	1	3	0
a10	0	1	38738	19477	1	44	81	55	70	1	1	1	3	3	15883	10955	1	47	81	59	65	1	1	0	2	0
a11	0	1	23774	12285	1	52	85	55	91	1	0	0	1	3	16764	10425	0	50	85	68	49	1	1	1	3	1
a12	0	1	35232	17624	0	37	74	61	35	0	2	1	3	3	18340	11917	1	39	74	45	83	0	0	1	1	1
a13	1	1	29997	15863	1	42	77	49	80	2	2	0	4	3	13206	8168	1	45	77	55	69	1	1	0	2	0
a14	1	1	29784	15451	1	35	77	52	60	1	1	1	3	3	19663	11046	0	45	77	63	44	0	2	2	4	0
a15	0	1	26872	14428	1	44	76	59	53	0	1	1	2	3	19207	11823	0	41	76	63	37	0	3	0	3	0
a16	0	1	30277	15562	1	39	80	52	68	0	0	2	2	3	26112	16426	1	44	80	47	92	2	0	3	5	1
a17	0	0	36487	20108	1	35	71	52	53	0	3	0	3	1	19811	12417	1	34	71	41	81	1	1	0	2	0
a18	1	1	25302	13193	0	39	76	60	43	0	2	1	3	3	29599	18839	1	36	76	43	83	0	0	3	3	0
a19	0	1	23203	12130	1	51	87	58	81	1	0	1	2	3	27358	16939	1	52	87	67	57	0	2	4	6	0
a20	0	1	40274	19481	1	42	84	50	81	2	0	0	2	3	26123	14729	1	45	84	51	85	2	0	1	3	1
a21	0	1	30058	14398	0	45	80	63	49	0	0	1	1	3	21809	13392	1	44	80	55	69	0	1	2	3	0
a22	0	1	42927	20199	1	37	81	49	73	1	1	0	2	3	22397	13001	1	45	81	45	100	0	0	2	2	1
a23	0	1	33490	16811	0	41	87	66	46	0	3	1	4	2	24428	15299	1	44	87	48	91	0	0	3	3	0
a24	1	1	46079	22791	0	46	86	69	43	0	3	1	4	3	23298	13662	1	44	86	56	71	1	1	1	3	1
a25	0	1	40129	20284	1	43	86	54	74	1	0	3	4	3	21425	13382	0	43	86	78	19	0	4	0	4	0
a26	0	1	33396	18576	0	44	89	68	47	1	2	1	4	3	24030	13335	1	51	89	64	66	2	0	0	2	0
a27	0	1	26264	13581	1	45	79	56	68	1	1	2	4	3	26926	14786	1	42	79	46	89	2	0	1	3	1
a28	1	1	32650	16251	1	55	94	67	69	1	0	0	1	3	24192	14175	0	60	94	80	41	1	2	3	6	0
a29	0	0	45313	23601	1	45	89	53	82	2	0	0	2	2	20183	13071	1	50	89	54	90	1	0	1	2	0
a30	0	1	40821	19487	1	45	87	60	64	0	0	2	2	2	28451	16234	0	46	87	71	39	2	2	1	5	0
**Mean**			33053	16628		43	81	58	62						22406	13574		44	81	55	71					
**SD**			6138	3070		4.9	5.6	7.0	17.3						4714	2657		5.3	5.6	10.3	20.5					
**CV%**			19	18		11	7	12	28						21	20		12	7	19	29					
**Median**										1.0	1.0	1.0	3.0	3.0								1.0	1.0	1.0	3.0	
**Sum%**	23	90			67												80									37
% bigger than L															6.3	5.9										

IPS IPS discontinuous = 0, continuous = 1 IPS/PCG IPS to PCG, disconnected = 0, connected = 1 GMWM Combined grey and white matter volume in mm^3^ GM Grey matter volume in mm^3^ SF or AS Sylvian fissure or Angular sulcus: short = 0 (i.e. sulcus <50% of the vertical height of the parietal lobe), long = 1 (sulcus >50% of the vertical height). Sup Superior limit of region (slice number in RView) Inf Inferior limit of region (slice number in RView) Top Top of SF or AS Pct Percentage height of SF or AS *G_ant Number of additional gyri anteriorly *G-sup Number of additional gyri superiorly *G_post Number of additional gyri posteriorly *G_Total Total number of additional gyri PISJ Prominence of PISJ: absent = 0, dimple = 1, vague = 2, prominent = 3 AS/IPS AS to IPS: unconnected = 0, connected = 1 SMG Supramarginal gyrus AG Angular gyrus PostCG Postcentral gyrus SupPL Superior parietal lobe L Left hemisphere R Right hemisphere SD Standard deviation CV% Coefficient of variation (SD/mean), expressed as a percentage Sum% Percentage of subjects having a score of 1

After the final round of checking, we extracted volumes for all parietal regions combining grey matter (GM) and white matter (WM).

MRIs were then segmented into three tissue classes using FSL FAST software 5.0.1 [53]. The resulting binary grey matter mask includes voxels where grey matter is the most likely out of the three tissue classes. This mask was then multiplied with the atlas images. Volumes for GM only were again extracted.

To account for effects of different head sizes between participants, the same intracranial volumes as in our previous studies [54,55] were used, determined via Exbrain 2.8.4 [56].

MRIs were spatially normalized to the Montreal Neurological Institute MNI152 reference space (MNI space) using SPM8 and the Unified Segmentation iterative spatial normalisation technique [57]. The above processes for extracting volumes were repeated in MNI space (which effectively standardises head size, [58]) to yield structure volumes in MNI space, and both for GM+WM and GM-only atlases (using the FSL FAST GM mask).

The main analyses were performed either in native space or using atlases spatially normalised with SPM8. A newer software version—SPM12—has been shown to yield different intracranial volumes [59] which were closer to manual reference volumes than those obtained with SPM8 for 1.5T scanners (cf. caveat in [60] for varying field strengths). We therefore repeated the volumetric analyses for SPM12 (via the Segment procedure) with standard settings.

### Creation of probabilistic atlases

We created probabilistic atlases by isolating each spatially normalised region in MNI space in turn and calculating the probability of each voxel belonging to that region. This process was performed for both GM+WM and GM-only atlases.

### Statistical analysis

The exploratory statistical analyses were carried out using R (http://www.r-project.org) with Spearman’s correlations and p-values thresholded at 0.005. All morphological characteristics and other variables in Tables 1 to 3 and 4 were paired to explore any correlations. We only report nontrivial correlations significant at the 0.005 level. In other words, if no correlations are reported in the tables, there were none that had p values below 0.005 among the demographic and morphometric / morphological variables listed in Tables 1 to 3 and 4.

**Table 4:** Volumes of parietal regions.

**Native space volumes in mm3**				
	**GMWM-Left**	**GMWM-Right**	**GM-Left**	**GM-Right**
**SMG**	28085 ± 5578* 23% of L PL CV 20%	25530 ± 5676 21% of R PL CV 22%	14078 ± 2697* 24% of L PL GM CV 19%	12765 ± 2775 22% of R PL GM CV 22%
**AG**	16109 ± 4824 13% of L PL CV 30%	17444 ± 4019 15% of R PL CV 23%	9844 ± 2653 17% of L PL GM CV 27%	10564 ± 2340 18% of R PL GM CV 22%
**postCG**	31405 ± 4080** 26% of L PL CV 13%	29278 ± 3531 25% of R PL CV 12%	12526 ± 1856* 17% of L PL GM CV 15%	11725 ± 1602 18% of R PL GM CV 14%
**supPL**	46008 ± 7978 38% of L PL CV 17%	46736 ± 7588 39% of R PL CV 16%	22160 ± 4116 38% of L PL GM CV 19%	22619 ± 3746 39% of R PL GM CV 17%
**MNI space volumes in mm3**				
	**GMWM-Left**	**GMWM-Right**	**GM-Left**	**GM-Right**
**SMG**	36194 ± 4985* CV 14%	33053 ± 6138 CV 19%	18172 ± 2221* CV 12%	16628 ± 3070 CV 18%
**AG**	20805 ± 6346 CV 31%	22406 ± 4714 CV 21%	12732 ± 3497 CV 27%	13574 ± 2657 CV 20%
**postCG**	41071 ± 3014** CV 7%	38636 ± 3609 CV 9%	16409 ± 1621* CV 10%	15490 ± 1832 CV 12%
**supPL**	58793 ± 5872 CV 10%	59677 ± 4985 CV 8%	28333 ± 3299 CV 12%	28917 ± 2606 CV 9%

Volumes are given as mm^3^ and also as a percentage of the ipsilateral parietal lobe in native space (results for MNI space were essentially identical).

PL = parietal lobe, SMG = supramarginal gyrus, AG = angular gyrus, postCG = postcentral gyrus, supPL = superior parietal lobe; GMWM = grey and white matter, GM = grey matter only; CV = coefficient of variation (standard deviation / mean, expressed as a percentage). * = bigger than contralateral (paired t test, * p<0.05, **<0.01)

### Multi-atlas label propagation

In earlier work we applied the previous version of the Hammers_mith atlas (with 83 regions) as a source of labels for multi-atlas label propagation using MAPER software, showing that automatic segmentation is feasible and accurate with this approach [61]. We carried out the same leave-one-out experiment (treating each subject image as a target, using the other 29 atlases as label sources) with the new Hammers_mith atlas version (95 regions, i.e. incorporating the new regions described here, and new insular subdivisions described in [55]). Using the manual labels as reference, we assessed the quality of the generated labels by determining overlap and volume aberration. We report here the results for the new parietal lobe regions.

## Results

In this section, we first present and discuss the morphological characteristics of the parietal lobe (Tables 2 and 3) in relationship to the participants’ demographic and brain variables (Table 1).

**Fig 2:**
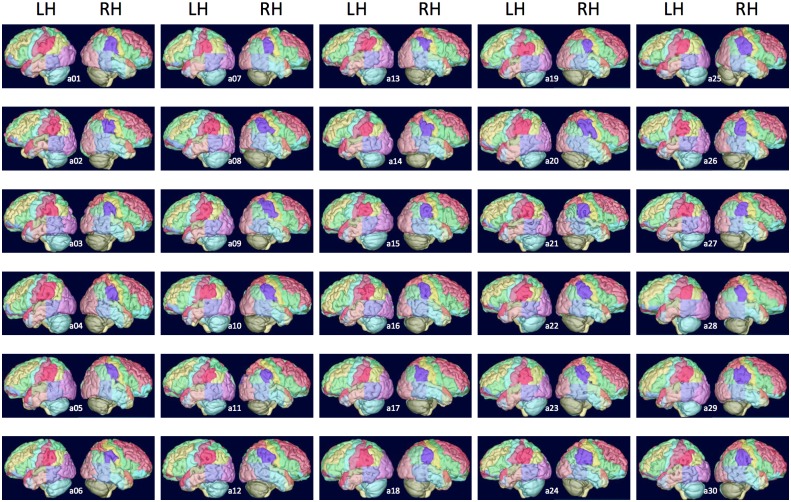
Lateral surface rendering of all sixty hemispheres. Compare with morphological characteristics per hemisphere in Tables 2 and 3.

We then present the volumes of the four parietal regions investigated (SMG, AG, postCG and supPL) for combined GMWM and GM only in native as well as MNI space (Table 4) and illustrate segmentation of all 60 hemispheres (Fig 2).

We then describe the relationships between the morphological characteristics and other variables listed in Tables 1 to 3 across participants (Tables 5 to 9).

**Table 5:** Intraregional / intrahemispheric additional gyri (G*).

	**Right SMG**	p	r	**Left SMG**	p	r
Total *G	SMG_R_*G_sup	0.002	0.5	SMG_L_GMWM	0.001	0.6
				SMG_L_GM	<0.001	0.7
				SMG_L_*G_ant	0.005	0.5
				SMG_L_*G_sup	0.002	0.5
Superior *G	SMG_R_SF	0.002	-0.5	SMG_L_SF_top	0.003	0.5
	SMG_R_SF_pct	0.001	-0.6	SMG_L_SF_pct	0.001	-0.6
Anterior *G	SMG_R_SF_pct	<0.001	0.6			
Posterior *G				SMG_L_SF	0.005	0.5
	**Right AG**	p	r	**Left AG**	p	r
Total *G	AG_R_AS_pct	0.003	-0.5			
	AG_R_*G_post	<0.001	0.6			
	SMG_R_*G_sup	0.002	-0.5			
Superior *G	AG_R_AS	<0.001	-0.7	AG_L_AS_pct	0.003	-0.5
	AG_R_AS_top	<0.001	0.7			
	AG_R_AS_pct	<0.001	-0.9			

Relationships between the morphological characteristics and other variables listed in Tables 1 and 2. Spearman’s correlations.

G*: number of additional gyri; p: p value (Spearman correlations); r: rank correlation coefficient.

Nomenclature: “SMG_R_*G_sup” stands for the number of additional gyri lying superiorly to the right SMG; “SMG_L_GMWM” stands for the volume of the left SMG in MNI space considering both grey and white matter, etc.

GMWM Combined grey and white matter volume in mm^3^ GM Grey matter volume in mm^3^ SF or AS Sylvian fissure or Angular sulcus: short = 0 (i.e. sulcus <50% of the vertical height of the parietal lobe), long = 1 (sulcus >50% of the vertical height). Sup Superior limit of region (slice number in RView) Top Top (superior end) of SF or AS Pct Percentage height of SF or AS *G_ant Number of additional gyri anteriorly *G_sup Number of additional gyri superiorly *G_post Number of additional gyri posteriorly SMG Supramarginal gyrus AG Angular gyrus L Left hemisphere R Right hemisphere

**Table 6:** Symmetry.

	**Left SMG**	**p**	**r**	**Left AG**	**p**	**r**
**Superior limit**	SMG_R_sup	0.004	0.5	AG_R_sup	<0.001	0.7
	AG_R_sup	<0.001	0.6			
**Inferior limit**	SMG_R_inf	<0.001	0.8	SMG_R_inf	<0.001	0.8
	AG_R_inf	<0.001	0.8	AG_R_inf	<0.001	0.8
	**Left SupPL**					
**GMWM**	supPL_R_GMWM	0.001	0.6			
	supPL_R_GM	<0.001	0.6			
**GM**	supPL_R_GMWM	0.002	0.6			
	supPL_R_GM	<0.001	0.7			

Relationships between the morphological characteristics and other variables listed in Tables 1 and 2. Spearman’s correlations.

p: p value (Spearman correlations); r: rank correlation coefficient.

GMWM Combined grey and white matter volume in mm^3^ GM Grey matter volume in mm^3^ Sup Superior limit of region (slice number in RView) Inf Inferior limit of region (slice number in RView) SMG Supramarginal gyrus AG Angular gyrus SupPL Superior parietal lobe R Right hemisphere

**Table 7:** Intrahemispheric correlation.

Left hemisphere		p	r
IPS connected to PCS: no = 0, yes = 1	AG_L_AS_top	0.003	0.5
	AG_L_*G_Total	0.002	0.5
PISJ prominence	PostCG_L_GM	0.001	-0.6

Relationships between the morphological characteristics and other variables listed in Tables 1 and 2. Spearman’s correlations.

G*: number of additional gyri; p: p value (Spearman correlations); r: rank correlation coefficient.

Nomenclature: “AG_L_*G_Total” stands for the total number of additional gyri in the left angular gyrus.

IPS IPS discontinuous = 0, continuous = 1 PCS Postcentral sulcus GM Grey matter volume in mm^3^ Top Top (superior end) of AS *G_Total Total number of additional gyri PISJ Primary intermediary sulcus of Jensen. Prominence of PISJ: absent = 0, dimple = 1, vague = 2, prominent = 3 AG Angular gyrus PostCG Postcentral gyrus L Left hemisphere

**Table 8:** Interhemispheric correlation.

Left hemisphere		p	r
SF percentage height	PostCG_R_GMWM	0.003	0.5
AS to IPS: unconnected = 0, connected = 1	AG_R_AS	0.001	-0.6

Relationships between the morphological characteristics and other variables listed in Tables 1 and 2. Spearman’s correlations.

p: p value (Spearman correlations); r: rank correlation coefficient.

Nomenclature: “PostCG_R_GMWM” stands for the volume of the right postcentral gyrus in MNI space considering both grey and white matter, etc.

GMWM Combined grey and white matter volume in mm^3^ SF Sylvian fissure AS Angular sulcus IPS Intraparietal sulcus AG Angular gyrus PostCG Postcentral gyrus R Right hemisphere

**Table 9:** Other correlations.

Left PISJ		p	r
PISJ prominence	Age	0.004	0.5
**ICV**			
ICV	Gender (male = 0, female = 1)	<0.001	-0.7
**Brain**			
Brain volume	Gender (male = 0, female = 1)	<0.001	-0.7

Relationships between the morphological characteristics and other variables listed in Tables 1 and 2. Spearman’s correlations.

p: p value (Spearman correlations); r: rank correlation coefficient.

ICV Intracranial volume PISJ Primary intermediary sulcus of Jensen. Prominence of PISJ: absent = 0, dimple = 1, vague = 2, prominent = 3

**Fig 3:**
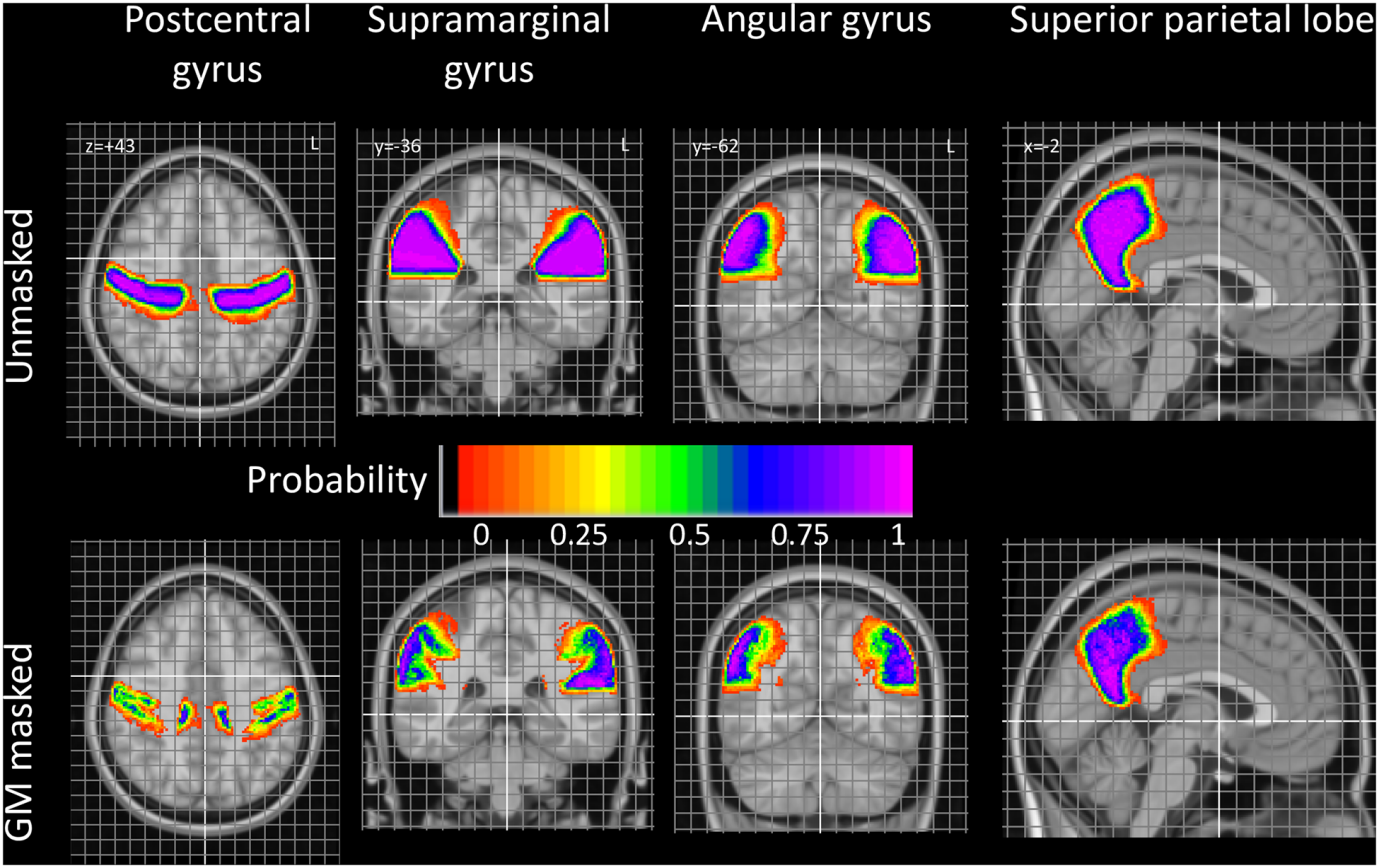
Summary pictures. Taken from detailed, finely spaced probabilistic maps (for full maps, see S2 File and www.brain-development.org).

**Fig 4:**
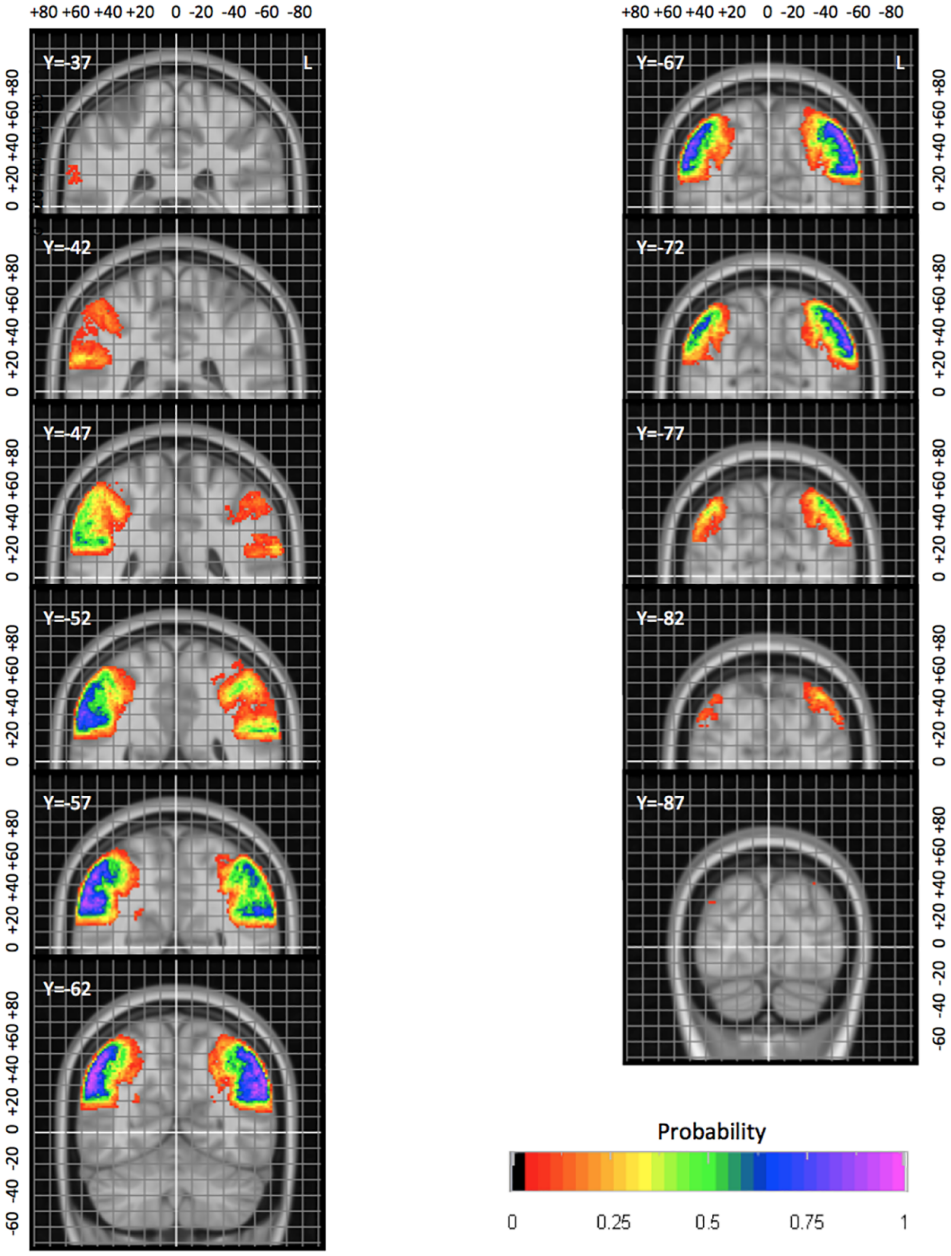
Example of a more detailed probabilistic map. AG in coronal orientation with 5 mm spacing between slices. The position of the MNI space origin near the anterior commissure is highlighted in white. The grey grid is spaced by five millimetres. Coordinates are in millimetres in MNI space relative to the origin. L, left.

Subsequently, we present the probabilistic atlases for all four regions (Figs 3 and 4; S2 File).

Finally, the results of using the manual segmentations for automatic segmentation via a multi-atlas registration and label fusion method are shown.

### Morphological characteristics (Tables 1 to 3, Fig 2)

Subject demographic and brain characteristics are shown in Table 1.

Details of morphological characteristics of the 60 hemispheres are shown in Table 2 (left parietal lobe) and Table 3 (right parietal lobe). For all variants in Tables 2 and 3, a graphical illustration of those characteristics is provided in Fig 2.

We observed a PISJ in all but one hemisphere (59/60), even if it was only present as a dimple in four left and two right hemispheres (cf. for illustration the protocols in S2 File, and notably Figs SMG-15 (prominent PISJ), SMG-16 (vague PISJ), and SMG-17 (PISJ only present as a dimple). As the PISJ was used as the boundary between SMG and AG, this allowed the allocation of additional gyri to SMG or AG. Such gyri, additional to the classical horseshoe shape, were frequently present, with a median of three for SMG and two to three for AG. These were anterior, superior or posterior to the main horseshoe shape.

Another major landmark of the parietal lobe, the IPS, was continuous in only 53% of left and only 23% of right hemispheres. It was usually connected to the precentral gyrus, i.e. in 87% of left and 90% of right hemispheres. The IPS was less frequently connected to the AS, i.e. in 20% of left and 37% of right hemispheres. In these cases, the AG does not form a continuous horseshoe shape wrapping around the AS.

### Volumes (Table 4)

All parietal regions combined (grey + white matter) were slightly (2%) larger on the left (121,607 ± 15,237 mm^3^) than on the right (118,987 ± 15,207 mm^3^; p < 0.03) in native space. As expected, a similar difference with overall larger volumes and less variability was found after spatial normalization (156,863 ± 8,686 mm^3^ on the left and 153,770 ± 10,561 mm^3^ on the right, p < 0.04). In MNI space, the entire parietal lobe represented 12% of the whole brain volume on each side for both grey and white matter, and 6% per side for the grey matter contribution.

Paired two-tailed Student’s t-tests of the MNI and native data (Table 4) showed that the volumes of postCG as well as SMG were significantly greater in the left than in the right hemispheres. The combined grey and white matter volumes for the postCG showed the greatest interhemispheric difference. No significant interhemispheric difference was present for the AG or the supPL.

Intersubject variability was highest for SMG and AG, and lowest for postCG.

The SPM12 volumes were very slightly larger (on average under 1%, and never more than 1.6%), a discrepancy that is in line with the small differences between the templates (tissue probability maps) used by the two SPM versions. The variance was near identical.

### Relationships between morphological characteristics (Tables 5 to 9)

The main observations concerned the number of additional gyri intraregionally (Table 4). For both the Sylvian fissure (SF) in SMG and the Angular sulcus (AS) in AG, the number of additional gyri lying superiorly to their main gyrus (commonly horseshoe-shaped) was negatively correlated with a measure of their length, i.e. the shorter the fissure/sulcus, the more additional gyri were present. This was true for both the left (L) and right (R) hemispheres and all results were significant (p≤0.005).

Other correlations also indicated a relationship between sulcal morphology and presence of additional gyri: Within the L-SMG, the length of the SF was positively correlated with the number of additional gyri posteriorly whereas in the R-SMG, the percentage vertical height of the SF was positively related to the number of additional gyri anteriorly.

For both SMG and AG, some of the numbers for anterior / superior / posterior additional gyri correlated with the total number of additional gyri (Table 5). Given the similar distribution of additional gyri across the three directions (Tables 2 and 3), this is expected.

There was also a relationship between total size and presence of additional gyri which only reached multiple-comparison corrected significance in the left SMG (Table 5). A *post hoc* comparison (Table 10) showed a similar relationship in the other inferior parietal regions. For the AG, this was similar on right and left, with about 20% of the variance in number of additional gyri explained by the volume of the region. The SMG was remarkable for having a much weaker relationship between these two variables on the right, but a much stronger relationship on the left, where volume explained up to 42% of the variance in additional gyri in this largely right-handed sample.

**Table 10:** Correlations between size of region (GMWM and GM) and number of additional gyri (*G).

	L SMG		L AG		R SMG		R AG	
	GMWM	GM	GMWM	GM	GMWM	GM	GMWM	GM
**p**	<0.0006	<9×10^−5^	<0.03	<0.02	<0.22	<0.06	< 0.02	<0.02
**r**	0.59	0.65	0.42	0.45	0.23	0.35	0.44	0.44

There was an overall tendency for the morphological characteristics to be symmetrical (Table 6). For example in the supPL, the volumes of grey and white matter were positively and significantly correlated across the hemispheres.

A certain amount of cohesion within hemispheres was also noted, for example between the measurements for the superior and inferior limits of regions as well as for the top of their relevant sulci, as would be expected (see supporting information to Tables 5 to 9 in S1 File).

Some other, more unexpected, observations were found to be significant (Tables 7 and 8), e.g. that in the L hemisphere, the IPS was more likely to be connected to the PCS, the shorter the AS (Table 7). Cross-hemisphere correlation findings included a left AS that was more likely to be connected to the IPS when the right AS was short, and a positive correlation between the right postCG volume and the height of the left SF (Table 8).

Table 9 shows that the prominence of the PISJ at its junction with the IPS increased with age; this reached significance on the left side only (left, r = 0.51, p < 0.005; right, r = 0.25, p < 0.18). There was the expected relationship of gender (Table 9) and intracranial volume (p < 0.0002, r = -0.72) as well as gender and brain volume (p < 7 ×10^−5^, r = -0.67), reflecting women’s smaller body sizes.

Each of the correlations shown above was also significant to a similar level of probability in the native space, except for that between the left PISJ prominence and the left PCG GM volume.

### Probability maps

Example slices taken from the probability maps are shown in Fig 3. The probability map for the AG is shown in greater detail in Fig 4; such detailed maps are available for all structures as supplementary material and on www.brain-development.org [for reviewers: http://soundray.org/hammers-n30r95/].

The probabilistic maps allow a number of observations. For example, the physiological brain torque, with structures in the right hemisphere being positioned more anteriorly than their counterparts on the left, is evident particularly for all four structures (see e.g. postCG in Fig 3, AG in Fig 4; full probabilistic maps in the supplementary material (supplementary S1 File) and at www.brain-development.org). Right/left positional difference can reach around 10 mm for certain probabilities (Fig 4), i.e. coordinates in one hemisphere cannot simply be mirrored onto the other hemisphere for images spatially normalized to asymmetrical templates. Sulcal variability can also be inferred: the anterior boundary of the postCG, i.e. the central sulcus, is far less variable than the posterior boundary, i.e. the postcentral sulcus. Other boundaries with remarkably little spatial variability in MNI space are the parietooccipital fissure and cingulate sulcus (Fig 3, superior parietal lobe).

### Automatic segmentation of the parietal lobe: Accuracy

The agreement between labels generated with MAPER and the manual reference labels was generally strong, with large overlaps (mean Jaccard index across 8 regions 0.69; equivalent to a Dice index of 0.82) and small volume aberrations (0.6%; Table 11). The Jaccard value for the AG on both sides were lower than expected for a region of this size. The automatic labels in this region pair tended to be biased toward the mean (overestimating the label when it was small compared to the group mean and underestimating it when it was large). The results for postCG, supPL, and SMG were in line with expectations, as we previously found similar values of agreement for other regions that are comparable in size, shape, and variability.

**Table 11:** Multi-atlas label propagation results.

Code	Region	Side	Manual volume	Automatic (MAPER) volume	Jaccard index	SD	CV%	Volume error (%)
32	AG	L	16109	14956	0.58	0.09	16	0.23
33	AG	R	17444	16361	0.54	0.09	16	-0.84
60	postCG	L	31404	31342	0.72	0.07	9	0.08
61	postCG	R	29277	29031	0.71	0.05	7	-0.35
62	SupPL	L	46008	46456	0.77	0.05	7	2.25
63	SupPL	R	46735	47170	0.78	0.04	5	1.73
84	SMG	L	28091	27700	0.73	0.05	7	0.80
85	SMG	R	25541	24977	0.68	0.07	11	1.13

Automatically generated labels were compared with manually drawn labels by overlap and volume. Code: Region number in the Hammers_mith atlases. L: left. R: right. Manual volume: mean manual reference volume in mm^3^. Automatic (MAPER) volume: mean volume of automatically generated label in mm^3^. Jaccard index: mean Jaccard index (n = 30). SD: standard deviation of Jaccard index. CV%: coefficient of variation (i.e. SD as a percentage of JC). Volume Error: Mean volume error as a percentage: [100*(automatic (MAPER) volume − reference (manual) volume) / reference volume] calculated per individual value pair, then averaged across 30 subjects.

## Discussion

The study continues our work on multi-subject atlases of the human brain and presents a detailed investigation of the macroscopic features of the parietal lobe. We contribute protocols for segmenting the inferior parietal region into the SMG and AG, complementing earlier protocols for postCG and supPL [16,19]. Volumetric and morphometric characteristics are provided, as well as probabilistic atlases in MNI space.

### Protocols

It is important to subdivide the human cortex for clinical and scientific investigation, and several methods are in use. Protocols aid consistent regional delineations and are essential for reproducibility and interlaboratory comparisons [62].

There are various kinds of information that can be used for creating atlases: macrostructural MR features, cytoarchitectonic features on post-mortem microscopy, hodology from diffusion tensor imaging, etc. Due to the presumed correspondence between cytoarchitectonic and functional subdivisions, *in vivo* delineation of cytoarchitectonic boundaries (corresponding to Brodmann or other areas) would be desirable. Some studies have used the distribution of myelin content to define putative cortical areal boundaries [63–65]. These authors refer to the observation that the inferoparietal region is one of the last to be myelinated [66]. Cross-modal comparisons of myelin maps with resting state fMRI may also increase the understanding of the cortical mosaic [37,67]. Patterns of regional connectivity also provide clues to developmental and functional organization of the mammalian cortex [68,69].

Here we have opted for macroscopic delineation on 3D T1-weighted MRI, a common imaging modality that is routinely used in conjunction with fMRI, DTI, and PET studies. We continued the use of protocols defined by Hammers et al. (2003), based exclusively on features observable on routine morphological MR imaging, to identify four parietal regions, namely SMG, AG, postCG and supPL. We followed the majority of studies by using the PISJ to separate the SMG from AG. In our study we were able to identify the presence of a PISJ in a greater proportion of brains than in previous studies. This may be due to our use of a wider range of descriptions, from ‘present as a dimple’, through ‘present but vague’, to ‘prominent’ (see examples illustrated in the protocols, Figs SMG-15 to SMG-17 in S2 File, and all sixty hemispheres illustrated in Fig 2 and their morphological characteristics in Tables 2 and 3). It may also be due to progress in delineation software, notably with the option of viewing the cortical surface at any chosen angle in real time during delineation. As the position of the AS was more difficult to ascertain than the SF due to the well-known complexity of the passage of the STS [46], the most prominent sulcus was routinely chosen, ascending into the AG.

We frequently noted additional gyri for both AG and SMG (Tables 2 and 3, Fig 2). It is possible that AG and SMG are not uniform regions but could be further subdivided anatomically into more specific subregions. Based on the detailed morphometric descriptions provided here, future studies could explore whether any consistent morphology-function relationships exist, as has been found e.g. for the hand area in the cingulate / paracingulate sulcus [70].

### Descriptive statistics and comparison with literature values

Face validity of our results is provided by replication of well-known relationships, e.g. between gender and ICV and therefore brain size (Table 8). Figures of regional volumes and coefficients of variation (CV) are also in line with the literature [18,40,71–73].

More specifically, in native space our parietal lobe mean GM volume of left 59 / right 58 cm3 is nearly identical to the mean of the two other studies providing such volumes [40,74] (left 59 / right 57 cm3); however we had noticed ~30% lower volumes in the older study [40] previously [75]. Correspondingly, for the different set of studies for which native total (i.e. GM+WM) parietal lobe volume are available [74,76], our volumes of left 122 / right 119 cm3 are around a quarter higher (ratio ~1.25) than the literature mean of left 96 / right 96 cm3. This may be due to protocol differences. For example, protocol illustrations indicate that the study with the smallest volume may have excluded the part of the parietal lobe posterior to the cingulate gyrus and anterior to the parieto-occipital fissure (supplementary material p. 99 in [76]).

Protocol differences are also evident for the parietal lobe regions. SMG, AG, postCG and supPL are available in [74,76]; in [72] volumes for SMG, AG and postCG but not supPL are provided. The ratio of our regional volumes to the literature volumes is higher than the overall lobar ratio of ~1.25 for SMG and postCG (around 1.5) and supPL (including precuneus; around 1.4) but lower for AG (around 0.95). The latter is driven by a very wide variability between studies, with a between-studies coefficient of variation of nearly 50% and a ratio of biggest [74] to smallest [72] estimates approaching 3.

While the studies providing total volumes were different from those providing GM volumes, precluding direct comparability, GM volumes tended to be more comparable between literature studies and our results, with ratios very close to 1 except for the AG which was about 20–25% smaller than literature means in our study. This should not be read as evidence for smaller variability when only the GM portion of volumes is assessed: the coefficient of variation across studies for all regions combined was more than three times higher than that for GM+WM combined (approximately 30% versus 8%).

Finally, it should be noted that sample sizes and compositions were different between studies. We studied 30 (15F/15M) brains of healthy participants aged 20–54; others used 10 subjects of undisclosed gender and age from a mixed population of healthy persons and those with various psychiatric conditions [40]; 14 controls (2F/12M) with age 18–55 as inclusion criterion [72]; 40 healthy controls (20F/20M) aged 19 to 39 [76]; or 59 healthy controls (24F/35M) aged 24±5 years [74].

All three factors (protocol differences; image processing differences and in particular tissue class segmentation; sample composition) will contribute to the variability seen in the literature, with protocol differences likely to play the biggest role.

We note in passing that the ratio of normalised to native total parietal lobe volumes changes between software versions. For SPM99 [58], it had been 1.45; for SPM5 (different data, [76]), it was 1.34; and this study, it was 1.29 for SPM8 and 1.30 for SPM12. Therefore, spatial normalization methods should always be described in full detail. Our ratios presented here may help in the comparison of data with different software versions, and indeed the native Hammers_mith atlases (www.brain-development.org) can be used for calibration of future combinations of normalization software and template spaces.

Between-subject variability, measured as the CV, was lower for the primary sensory region, i.e. the postCG, than for the other regions, as expected (Table 4). While our results in native space are presented uncorrected for ICV, spatial normalization to MNI space is, effectively, a normalization by total brain volume. We had quantified this previously [58]: between-subject coefficient of variation (CV) of all brain structures (approximating total brain volume) dropped from 11% in native space to 2% in MNI space. The CV of postCG and supPL was reduced following spatial normalization, quantifying the homogenizing effect of the procedure. The more pronounced reduction in CV for the supPL compared with AG and SMG may partly be due to the relative ease of spatial normalization along the outer boundaries of the brain and the pronounced hemispheric border, which acts as a major landmark through the high GM-CSF contrast.

The probabilistic maps allow an assessment of regional variability and will help put functional studies in context, e.g. through a probabilistic assessment of a functional activation being in a particular part of the PL. As mentioned above, they are complemented by histology-based, myelin-based, and hodological atlases.

Volumes were larger in MNI space; this is a known effect stemming from the MNI reference space being substantially larger than average brains [16,77].

It was interesting to find that the parietal lobe was slightly (~2%) larger on the left. We consider this to be driven by leftward asymmetries of the postCG and SMG, and it may well be related to the majority of subjects being right handed (left-hemisphere dominant). Previously, this slight leftward asymmetry has been reported by some authors [18,73], but no asymmetry has been found by others [71].

### Morphometric features

We explored the volumetry data for relationships between the anatomical features recorded for the 60 hemispheres from 30 adult brains. The descriptive data was tested for correlations. Expected correlations like the relationship between ICV and brain size translated into correlations between ICV and PL region size and served as a plausibility test. The results show clear correlations (negative and positive) between the number and distribution of gyri in relation to the major intraregional sulci, many of which have intuitive interpretations.

There was a clear negative correlation between the number of additional gyri superior to the horseshoe shapes of AG and SMG and the vertical extent of the major intraregional sulci. This may be purely a mechanical effect of limited distance between the dorsal end of the sulcus (SF or AS) and the IPS. However, there may also be a relation with the developmental course of the formation of the gyri and that of the sulci. These would be interesting aspects to study for the future, particularly in developing brains. This will be rendered possible by emerging techniques for imaging the human brain in utero [78]. In the SMG, when the SF was short, most of the additional gyri tended to be superior or anterior, whereas in the AG, when the AS was short, they lay posteriorly. This may indicate a structural role of the PISJ as a physical barrier between the SMG and AG, leading to gyral matter being more tightly packed on the external borders of the regions, that is, towards the PCG or the occipital lobe.

Purely mechanical factors may also have been involved in the observation that in the left hemisphere, the IPS was more likely to be disconnected from the PCG, the more dorsally the AS extended and the fewer total additional gyri were present in the AG. This may reflect a more anterior placement of the cortical matter in the SMG, causing a gyral bridge with the superior parietal cortex.

The observation of increased PISJ prominence with age, significant on the left side, could be due to brain shrinkage and was detected despite the restricted age range of our sample (20–54 years). Widespread increases in sulcal span with age have been reported [79].

The distribution of additional gyri may also relate to the angle of the SF or AS, in that an acute angle may produce more gyrification posteriorly, ventral to the sulcus. Similarly, the anterior-posterior direction of the PISJ may have affected the distribution of additional gyri. Both of these aspects were too complex to quantify due to the undulating nature of the sulci. Nevertheless, it was interesting to note that while the position of the additional gyri (anterior, superior or posterior) correlated with the total number of additional gyri, they did not correlate negatively with each other. If the correlations elicited had simply been related to the arbitrary assignment of the position of the SF or AS, then a negative correlation, e.g. between those lying anteriorly compared with those lying posteriorly, would have been expected.

The gyrification of the cortex is a growing area of research [80,81]. Although the present work has considered only structural aspects, recent studies increasingly suggest the importance of the role of gyrification in the mammalian brain and the effects of abnormalities of gyrification during developmental processes [42,79,82–88]. Methods to measure gyrification have been described [89]. Furthermore, the role of genetics in the development of brain structure is being studied across species [90–92], and very recently there have been indications of gene-related mechanisms underlying cortical gyrification, possibly species-specifically [82].

### Automatic segmentation of the parietal lobe via multi-atlas label propagation

The present work investigated morphological characteristics in the parietal lobes of 60 hemispheres. Thanks to existing methods exploiting the knowledge incorporated in manual delineations for automatically anatomically segmenting large numbers of brain images [48–50], it is possible to use the present atlases for the study of new control and disease cohorts. Future studies could hence also explore the correspondence between the current anatomical delineations and fMRI distributions of activity.

The leave-one-out experiment we carried out using MAPER software yielded the expected results for postCG, SupPL, and SMG, indicating that these regions can be automatically propagated to newly acquired images with a high level of accuracy. The automatic AG labels were somewhat less accurate than expected, showing relatively large intersubject variability and a volumetric bias toward the atlas mean. We will follow up on this result, as it adds to existing evidence that MAPER might be improved through measures that reduce bias toward the mean (e.g. atlas selection, [93]). Note that the AG has been found to be more variable between subjects than other parietal lobe regions in previous work, too. For example, Crespo-Facorro et al. [94] obtained coefficients of variation of over 50% for this region, as opposed to coefficients of variation between 13 and 26% for other parietal regions, despite segmentation after spatial standardization (of a mixed sample of ten subjects who were either healthy or had a variety of psychiatric diseases). In addition, there is evidence of larger than usual protocol heterogeneity, with the ratio of largest-to-smallest average volume across three studies [72,74,76] around 3 for the AG as opposed to between 1.1 and 1.8 for the other parietal regions.

## Supporting information

S1 File Complete probabilistic maps and supplementary table. (DOC)

S2 File Illustrated delineation protocol for the parietal lobe. (DOCX)
