# Supplementary material for: Gyri of the human parietal lobe: Volumes, spatial extents, automatic labelling, and probabilistic atlases
Source: PLoS One. 2017 Aug 28;12(8):e0180866. doi: 10.1371/journal.pone.0180866 (PMC5573296; doi:10.1371/journal.pone.0180866)
Supplement: S1 File — (DOC) [file pone.0180866.s001.doc]

**Supplementary material: Probabilistic maps and additional table**

**Probabilistic maps**

The four region probabilistic maps are shown in detailed slices in the following. The 3D image files are available through [www.brain-development.org](http://www.brain-development.org/) (for reviewers: <http://soundray.org/hammers-n30r95/>). We first show unmasked probabilistic maps, followed by probabilistic maps with grey matter only.

**Supplement to** **Table 3: Cohesion within hemispheres** (correlation between positions of boundaries)

|  | **Right SMG** | p | **r** | **Left SMG** | p | **r** |
| --- | --- | --- | --- | --- | --- | --- |
| **Superior limit** | SMG_R_inf | 0.004 | 0.5 | AG_L_AS_top | <0.001 | 0.6 |
|  | AG_R_inf | 0.004 | 0.5 | SMG_R_inf | <0.001 | 0.7 |
|  | AG_R_AS_top | 0.002 | 0.5 | AG_R_inf | <0.001 | 0.7 |
|  |  |  |  | AG_R_AS_top | 0.001 | 0.6 |
| **Inferior limit** | AG_R_sup | <0.001 | 0.7 | AG_L_sup | 0.004 | 0.5 |
|  | AG_R_AS_top | 0.001 | 0.6 | AG_R_sup | 0.001 | 0.6 |
|  | **Right AG** |  |  | **Left AG** |  |  |
| **Superior limit** | AG_R_inf | <0.001 | 0.7 | AG_L_inf | 0.004 | 0.5 |
|  | AG_R_AS_top | 0.001 | 0.6 | AG_L_AS_top | <0.001 | 0.7 |
|  |  |  |  | SMG_R_inf | 0.001 | 0.6 |
|  |  |  |  | AG_R_inf | 0.001 | 0.6 |
| **Inferior limit** | AG_R_AS_top | 0.001 | 0.6 | AG_R_sup | 0.001 | 0.6 |

**Legend for Supplement to Table 3:** Relationships between the morphological characteristics and other variables listed in Tables 1 and 2. Spearman’s correlations.

Sup Superior limit of region (slice number in RView)

Inf Inferior limit of region (slice number in RView)

Top Top of SF or AS

SMG Supramarginal gyrus

AG Angular gyrus

L Left hemisphere

R Right hemisphere

A certain amount of cohesion within hemispheres (Supplement to Table 3) was noted, for example between the measurements for the superior and inferior limits of regions as well as for the top of their relevant sulci, as would be expected.
