# Supplementary material for: Gyri of the human parietal lobe: Volumes, spatial extents, automatic labelling, and probabilistic atlases
Source: PLoS One. 2017 Aug 28;12(8):e0180866. doi: 10.1371/journal.pone.0180866 (PMC5573296; doi:10.1371/journal.pone.0180866)
Supplement: S2 File — (DOCX) [file pone.0180866.s002.docx]

**Gyri of the human parietal lobe:**

**Volumes, spatial extents and probabilistic atlases**

**HM Wild^1,2^, RA Heckemann^1,3^, C Studholme^4^, A Hammers^1,5^**

**Appendix:**

**Protocols for supramarginal gyrus (SMG) and angular gyrus (AG) – for the protocols for postcentral gyrus and superior parietal lobe, see Gousias IS et al. Neuroimage 2008.**

**For the detailed probabilistic maps for the four parietal subregions: full regions and grey-matter masked (50% probability), see Supplementary Material (separate file).**

**Appendix: Protocols for SMG and AG**

Method

General considerations

1. The SMG and AG were drawn on the 30 atlases previously developed [1,2]. Coronal slices were used to delineate borders, with constant comparison between transverse and sagittal views for confirmation. Rviewqt was used to create a surface view from the segmented GM/WM data, and this was used as the arbiter for decisions in ambiguous situations, e.g. for identifying the PISJ.


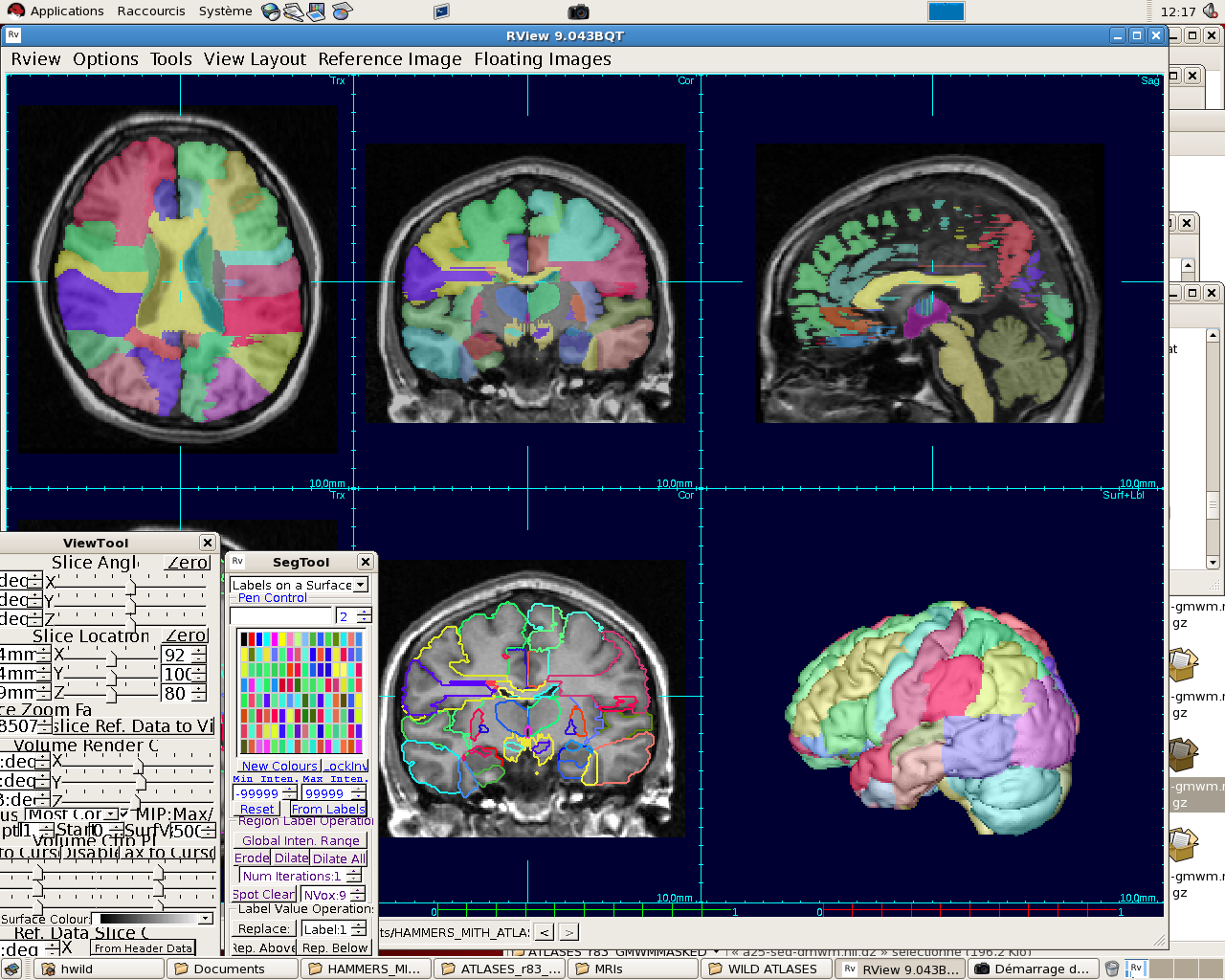


Figure Appendix-1. Example of a surface view used (atlas 25, left hemisphere - a25 LH)

1. The rviewqt software window layout consisted of simultaneous views of the MRIs, the masked surface view, and the atlas contours overlays. The tomographic images were displayed right on left side of the image and vice versa; the surface views presented a natural view.
2. The rviewqt colour scale was selected such that the same region in the two hemispheres had a different colour, and that colours were not similar to adjacent regions.
3. In the Hammers_mith atlases, odd numbers designate regions in the right and even numbers regions in the left hemisphere. SMG was allocated number 84 (left) and 85 (right). The AG retained numbers 32 (left) and 33 (right) originally assigned to the inferior parietal lobe.
4. Prior to outlining either SMG or AG, the position of the IPS/PISJ junction was found by moving the cross hairs and checking with the surface view. It was easiest to find the correct height on the transverse view first and then move the coronal slices.
5. The area anterior to the PISJ is labelled as SMG, leaving the previously labelled posterior area as AG.
6. Coronal slices were continually checked with other views, especially the surface view. Any apparent ‘spikes’ were included, rather than smoothed out, if they were consistent with other views so as not to lose anatomical information. ‘Spikes’ of label were usually due to the transition between the previously established protocols used with transverse sections compared to use of coronal sections.
7. In general, the orientation of the surface view was frequently adjusted as the shading on sulci and gyri made them easier to identify and estimate their prolongations. The change in orientation was also helpful when the surface view may have suggested the presence of a sulcus whereas the cross-sectional view showed it to be actually a smooth surface, or at least only a ‘bump’.
8. The total number of slices containing the region outlined (SMG /AG) was recorded.
9. Continual discussion between the main investigators enabled consensus decisions and avoided drift in protocol interpretation.
10. After the final segmentation was obtained, morphological features were noted for both SMG and AG.

**SUPRAMARGINAL GYRUS – PROTOCOL**

The supramarginal gyrus (SMG) is part of the parietal lobe, corresponding to Brodmann’s area 40 [3] (p. 140 and Figure 85, p. 131). It is involved in language skills and peripersonal perception. Anatomically, the SMG is bounded by the intraparietal sulcus (IPS), the postcentral sulcus (PCS), the position of the Sylvian fissure (SF) and the primary intermediate sulcus of Jensen (PISJ).

These reference boundaries were established using mainly brain atlases [4,5], brain mapping literature including our prior work [1,2], and LONI Protocols (http://cms.loni.ucla.edu/ncrr/protocol).

In general, the SMG forms a horseshoe shape around the SF (Figure SMG-1). Anteriorly it is often nestled into the concave bend of the PCS, creating one or sometimes two islands in coronal sections. Posteriorly, the PISJ separates the SMG from the angular gyrus (AG).


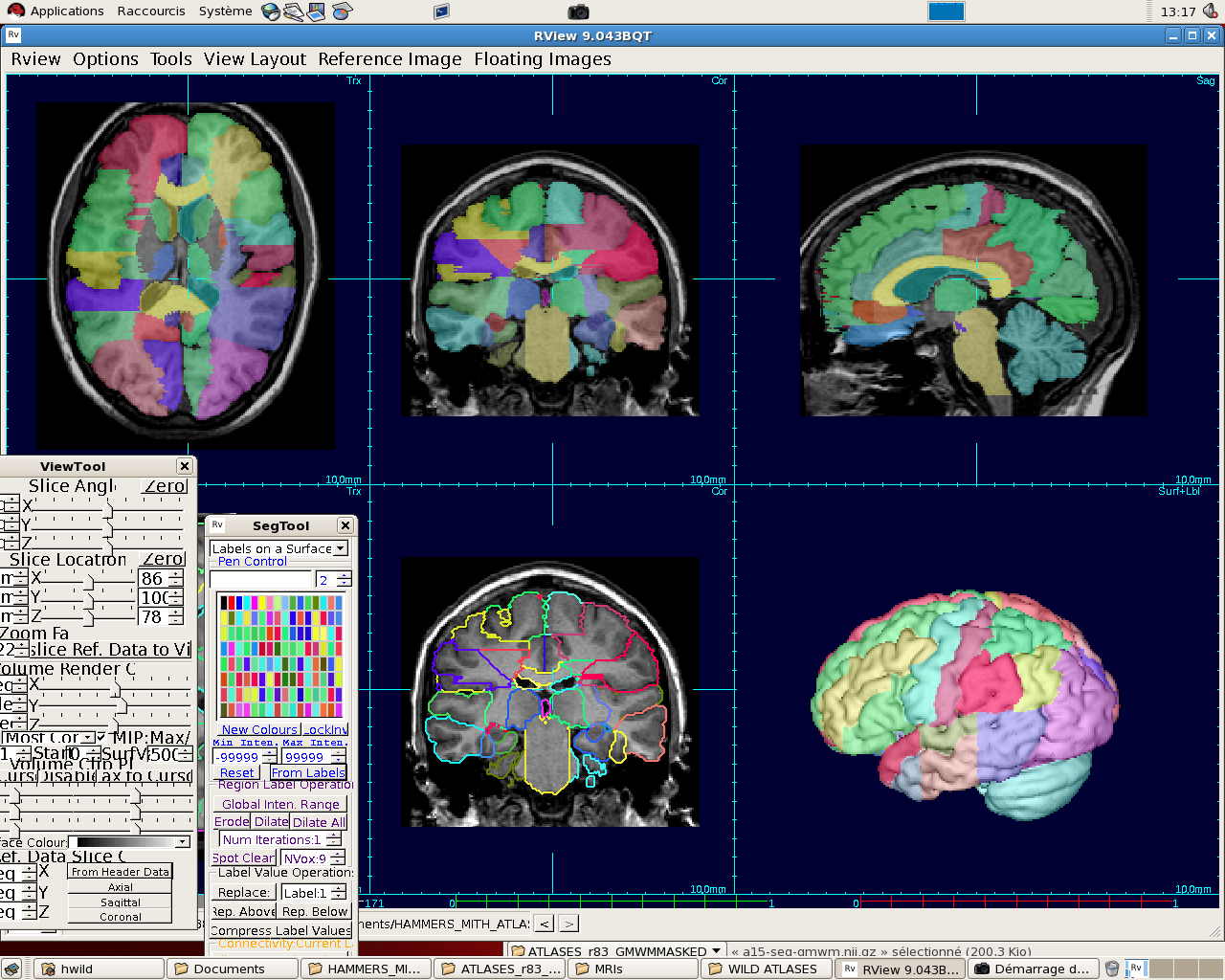

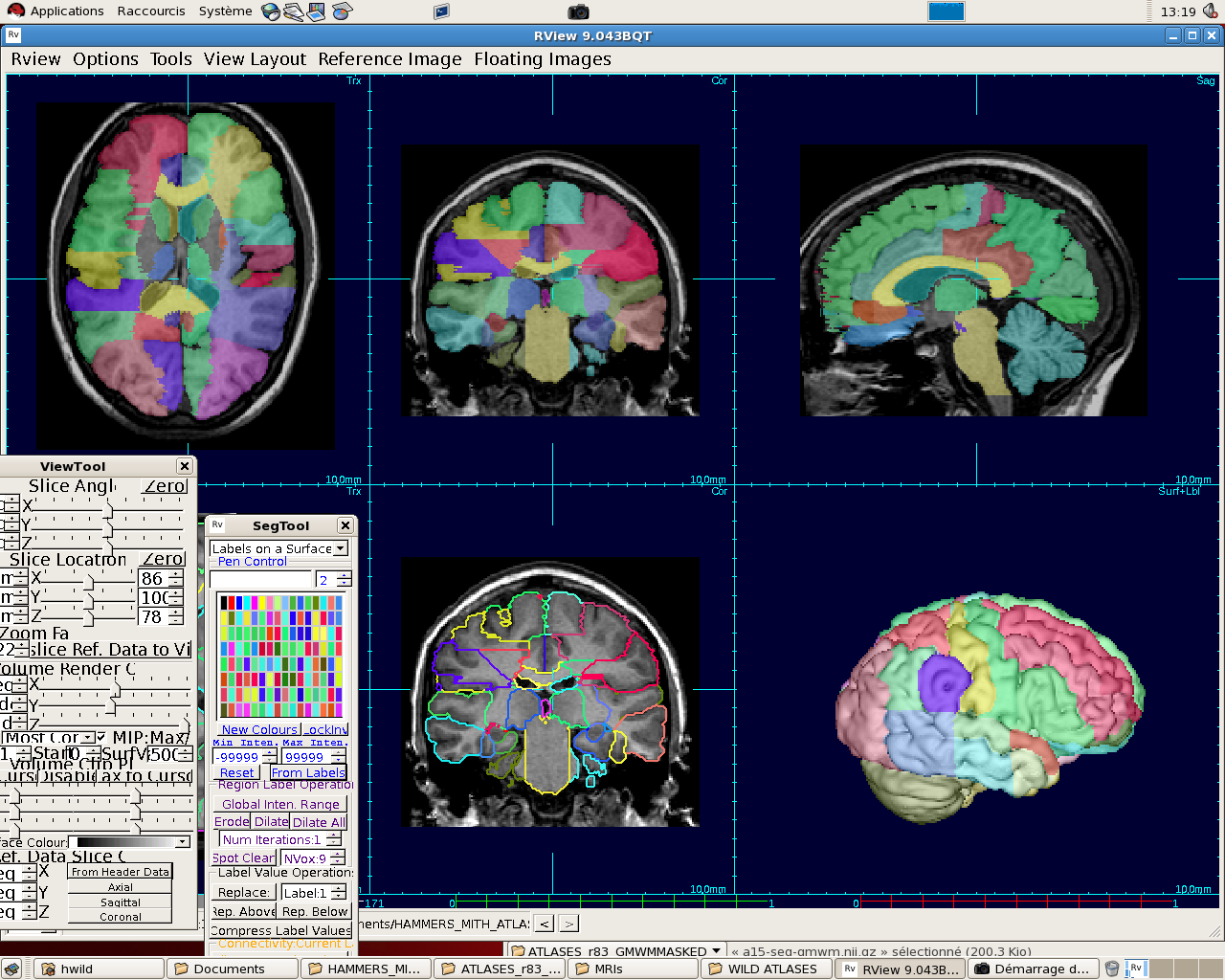


Figure SMG-1. Surface views of SMG. Hemispheres used; a15, left hemisphere (SMG in red) and right hemisphere (SMG in purple) (a15 LH, a15 RH)

The present protocol uses the boundaries of the inferior parietal lobe previously created during the work by [1,2]. Their brain atlases ([www.brain-development.org](http://www.brain-development.org)) delineate 83 regions of interest including the parietal lobe which had been divided into postcentral gyrus, superior parietal lobe, and inferior parietal lobe. The aim of this study was to divide the inferior parietal region further into the SMG and the AG.

**Definition of the PISJ as the boundary between SMG and AG**

Segmentation begins at the junction between the IPS and the PISJ which usually lies midway between the anterior and posterior borders of the inferior parietal lobe region and is best seen on the surface view. Sometimes this junction is clear, but it may also be vague or appearing only as a dimple. In the rare cases when it is completely absent, the starting point may be taken as where the vertical extension of the midpoint between the SF and the STS meets the IPS.


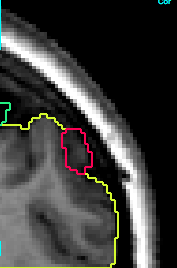


Figure SMG-2. Example of an SMG island (a01 LH)

If the PISJ is long and the coronal slice cuts through it at two points, an island will be formed, surrounded by CSF. In this case only the island is labelled SMG (Figure SMG-2). This also applies if development of the PISJ results in areas of AG being superior and inferior to the SMG label.

In these cases, the labelling continues to include the adjoining WM but only until the GM starts to separate from the underlying sulcus, at which point only the separated SMG island is labelled.

The dividing line between SMG and AG is drawn from the defined surface point to the most inferiomedial part of the SMG.


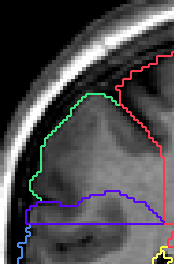
This dividing line is straight when the region to be crossed contains only WM. If the defined point at the surface lies at the beginning of a sulcus, then the dividing line follows the course of the sulcus to its deepest point before bending to then join the most inferiomedial part of the SMG (Figure SMG-3). This is the case even when the sulcus continues to bend dorsally.

Figure SMG-3 (a01 RH). The red line defining the boundary between SMG and AG follows the sulcus before being extended to the inferomedial boundary of the previously defined inferior parietal region.

If a neighbouring sulcus has to be crossed while drawing through the WM, the dividing line deviates accordingly following the sulcal groove, before resuming its straight course to the most inferiomedial part of the SMG (previously inferior parietal lobe).

Each slice is labelled to include all the GM and WM for that section.

In the following, the six boundaries are described similar to the protocols included in [1,2].

**Superior boundary = IPS**

This is usually clear from the surface view, especially when the orientation is varied to the most posterior angle. It is helpful to confirm the identity of the IPS by taking several transverse views, more dorsally and ventrally, which show the full extent of the sulcus.

Ono et al. [5] describe the IPS as being continuous in 28% of right hemispheres and 72% of left hemispheres, and split into two segments in the remainder.


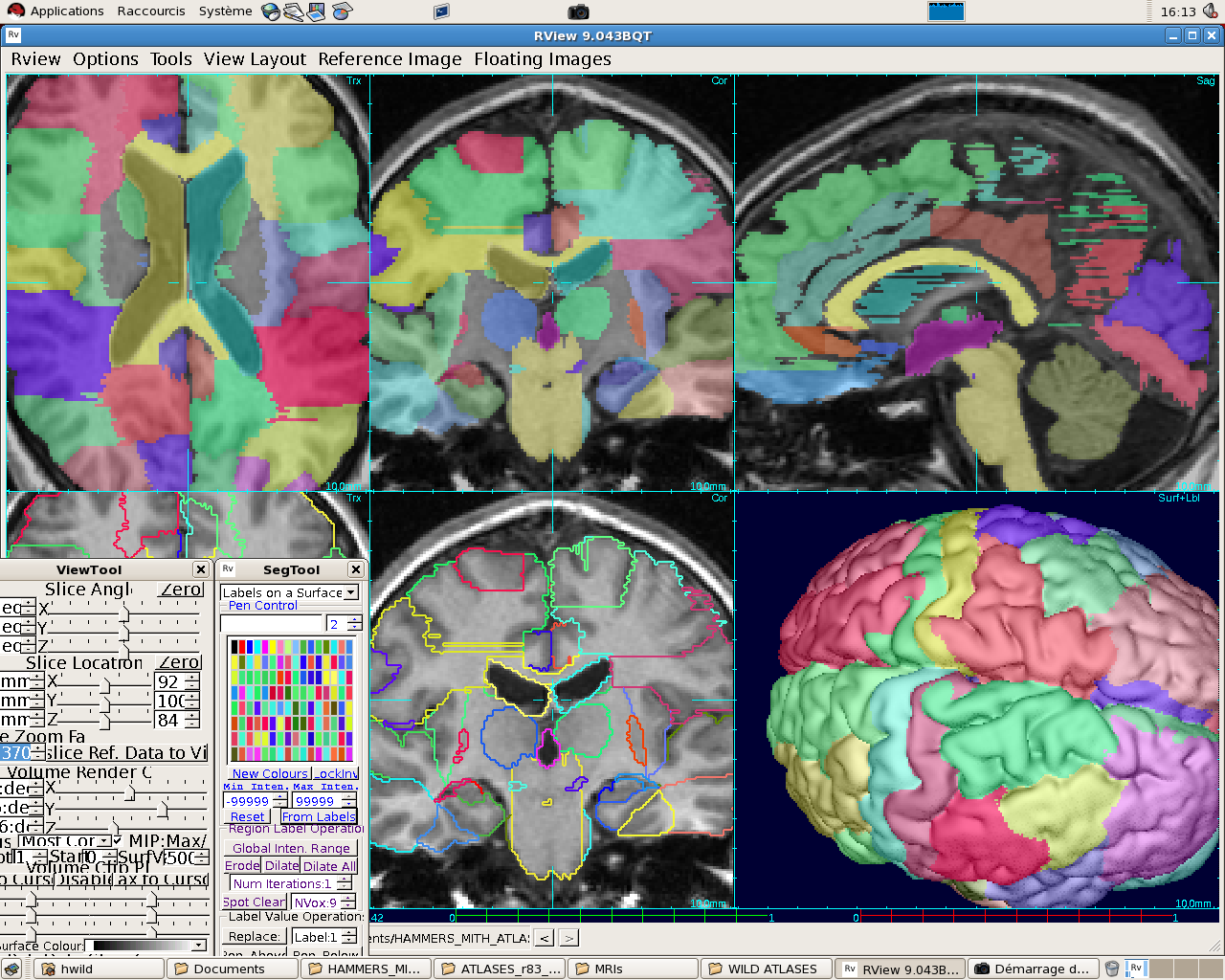


Figure SMG-4 showing a continuous IPS (a29 LH and RH)


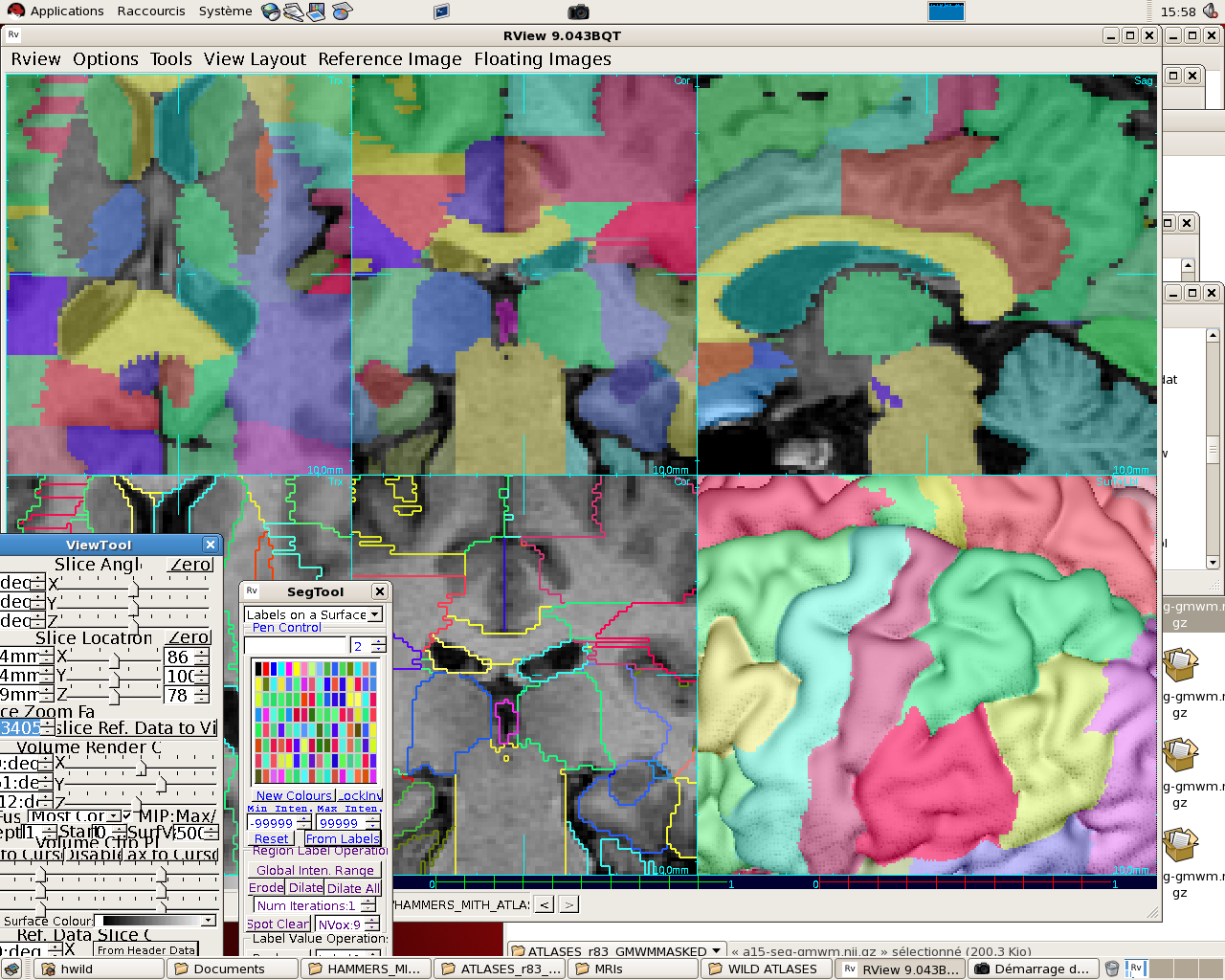


Figure SMG-5 showing a discontinuous IPS (a15 LH), necessitating the crossing of a gyrus to reach the PCS.

In some cases, the IPS was connected to the PCS (Figure SMG-6) and in other cases it was unconnected (Figure SMG-7).


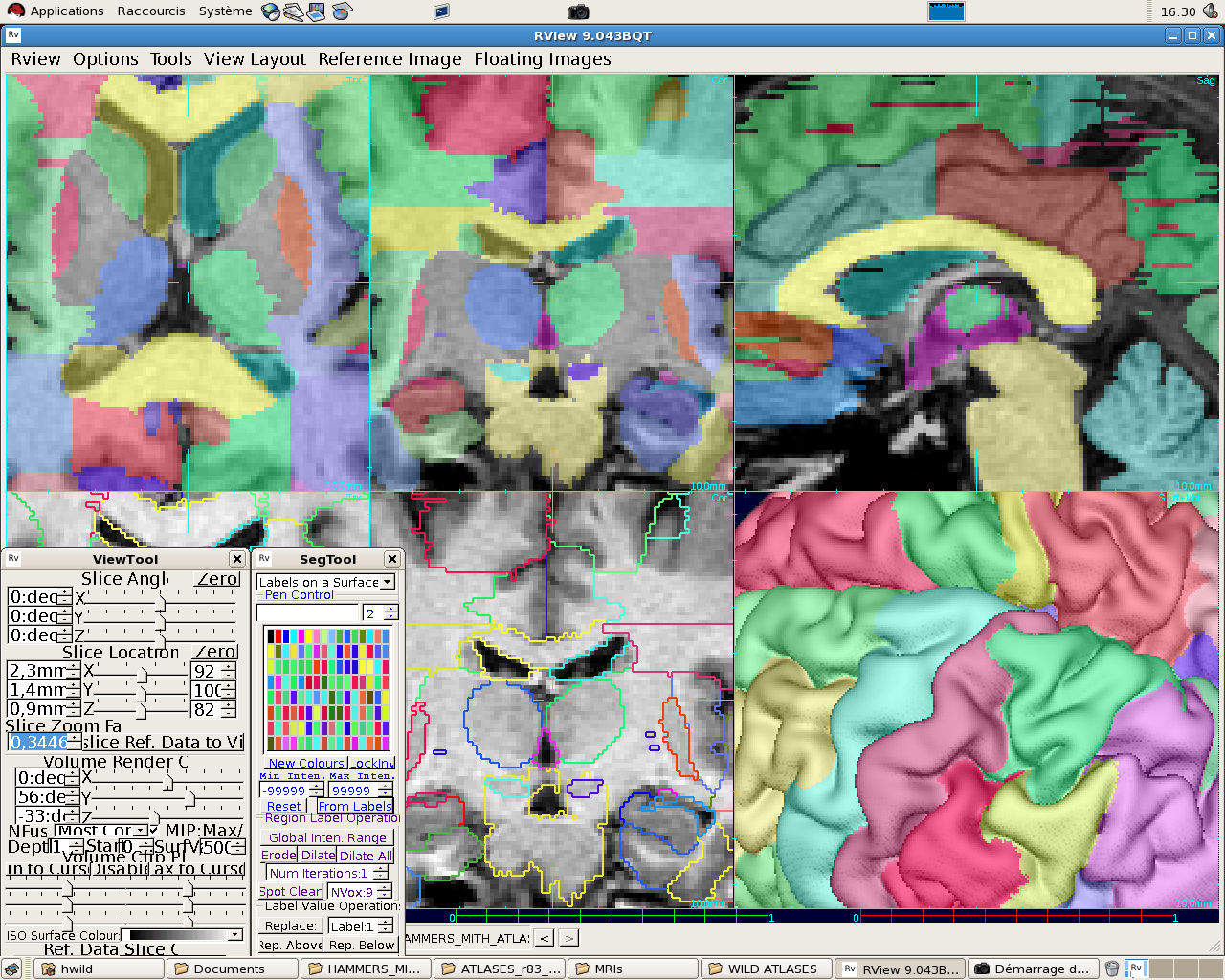


Figure SMG-6: IPS connected to PCS (a03 LH)


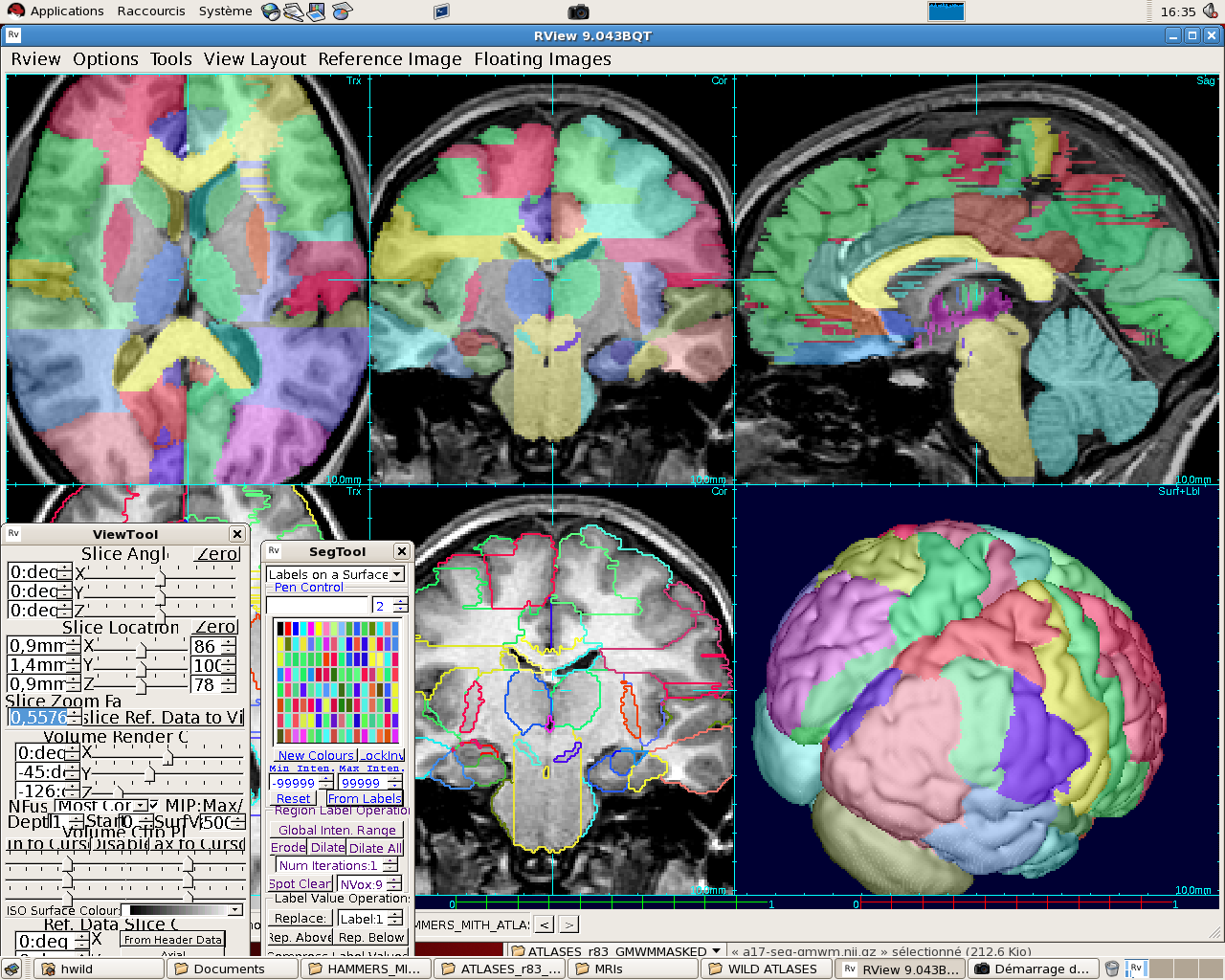


Figure SMG-7: IPS unconnected to PCS (a17 RH), necessitating crossing of a gyrus (arrow).

**Inferior boundary = Sylvian Fissure (SF)**

The SMG area is labelled to include the ascending part of the SF that it surrounds like a horseshoe. The labelling continues to the horizontal line defined by the previous protocol at a point midway between the SF and the STS.


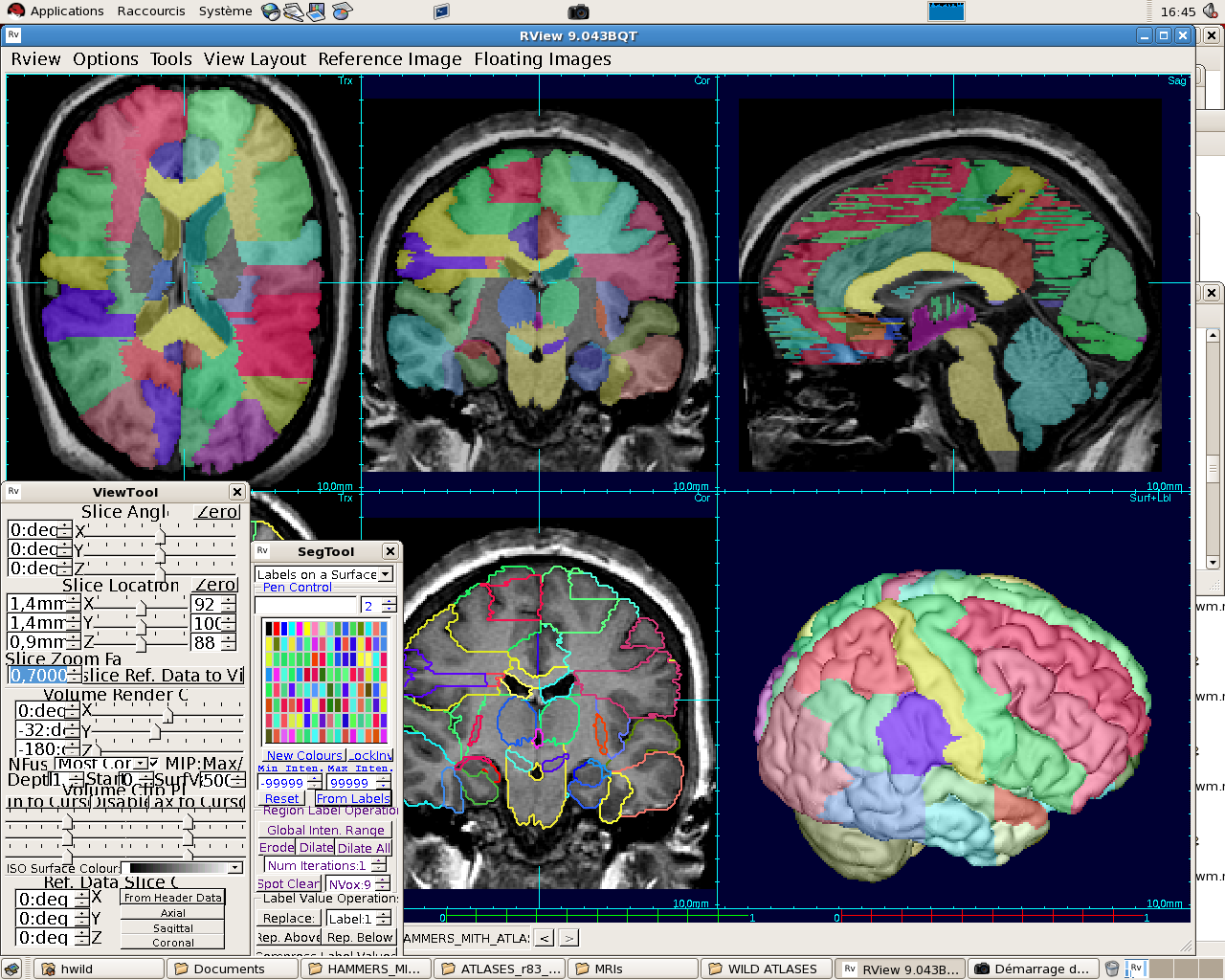


The SF was considered to be long if it ended above the vertical midpoint between the superior and inferior extent of the SMG, short if it did not (Figures SMG-8 and SMG-9).

Figure SMG-8: Long SF (a19 RH)


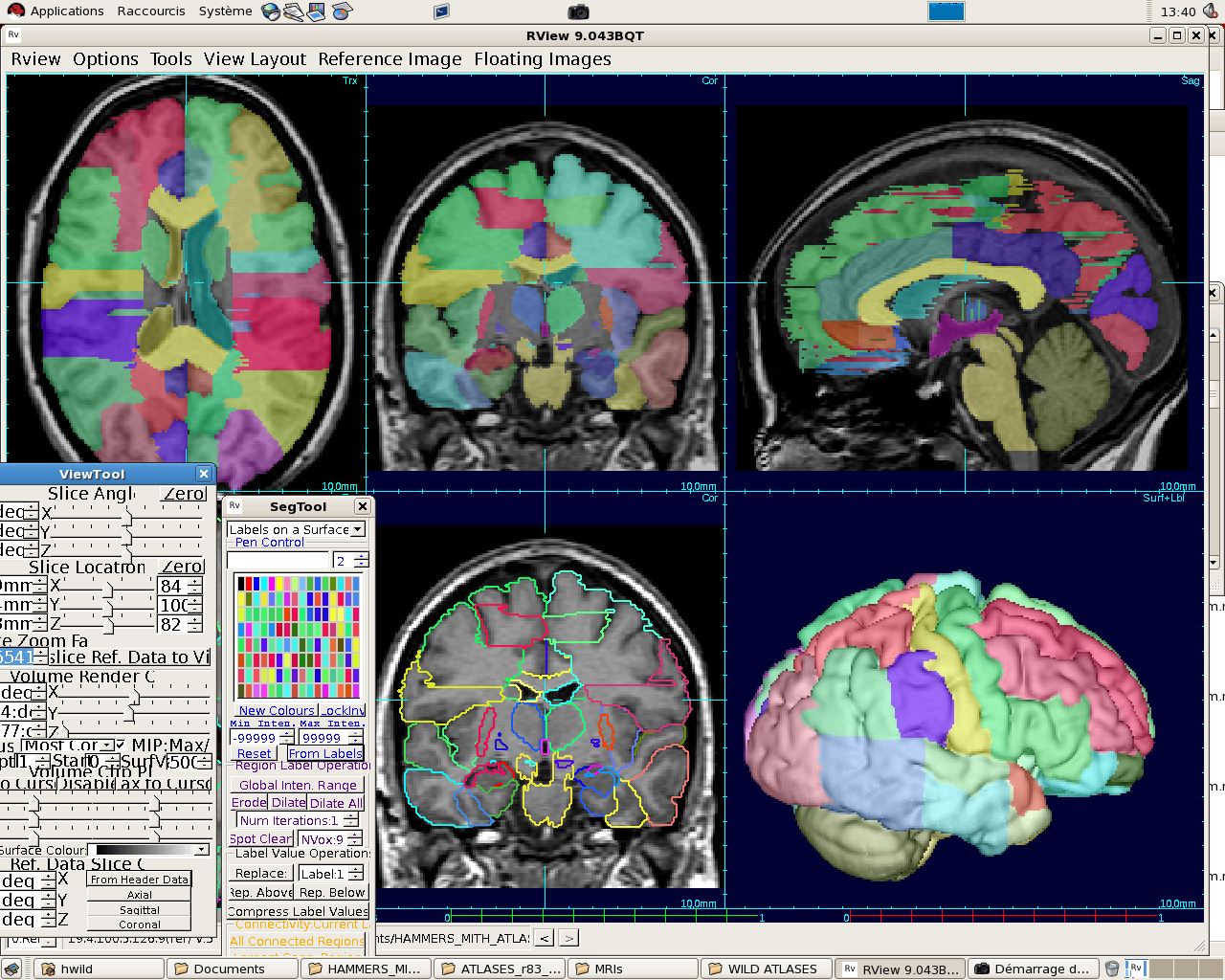


Figure SMG-9: Short SF (a07 RH)


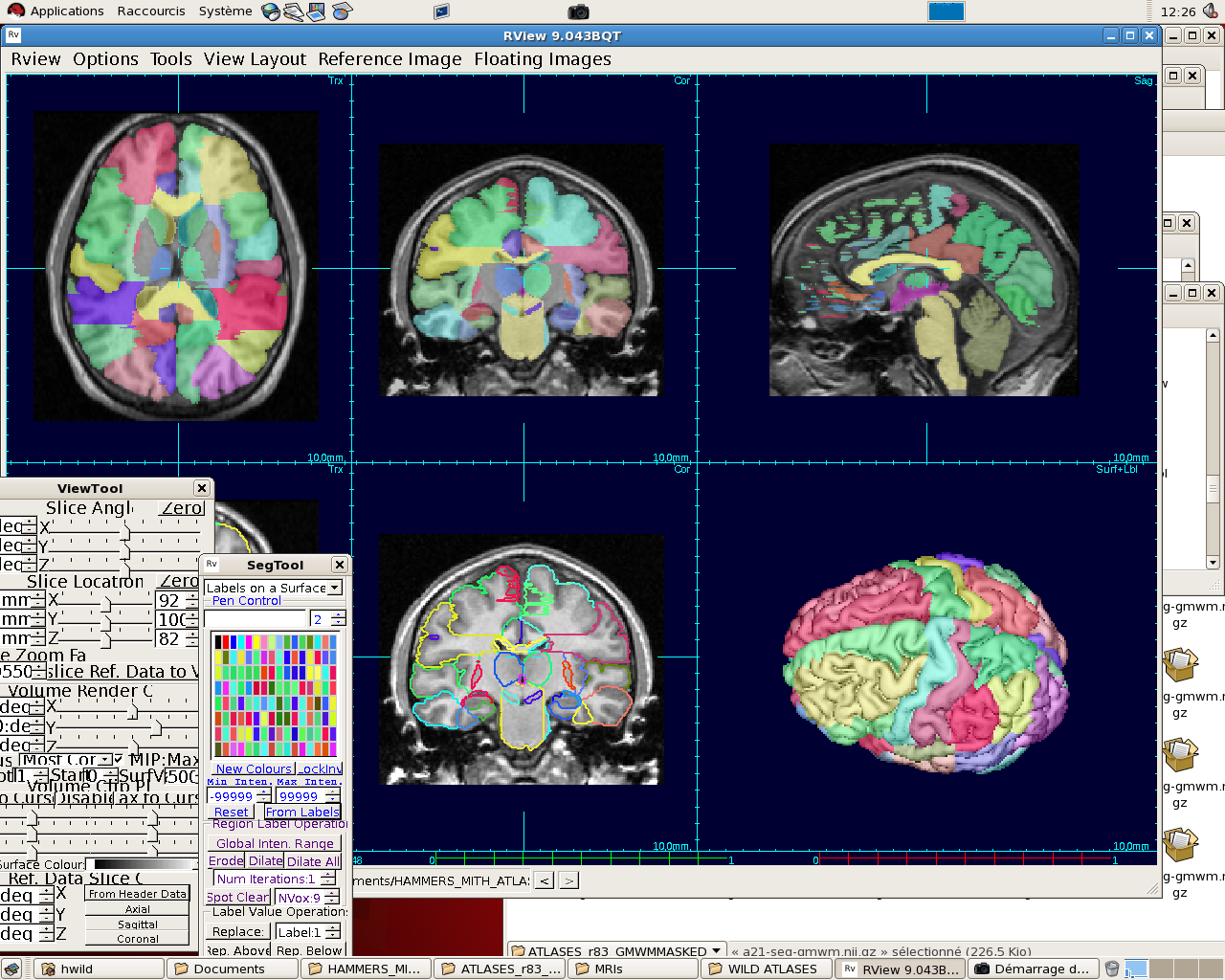


Figure SMG-10: Additional gyri (a21 LH)

This measure is useful because it may be that when the SF is short the presence of one or more additional gyri inferior to the IPS is more likely. The presence of an additional gyrus was therefore recorded to see if there was any correlation between these observations (Figure SMG-10)

**Anterior boundary = PCS**

The PCS posterior border had been defined as the deepest sulcus posterior to the central sulcus, usually resulting in one gyral width. The presence of any SMG island(s) nestled in the PCS (as described above) was recorded (Figure SMG-11).


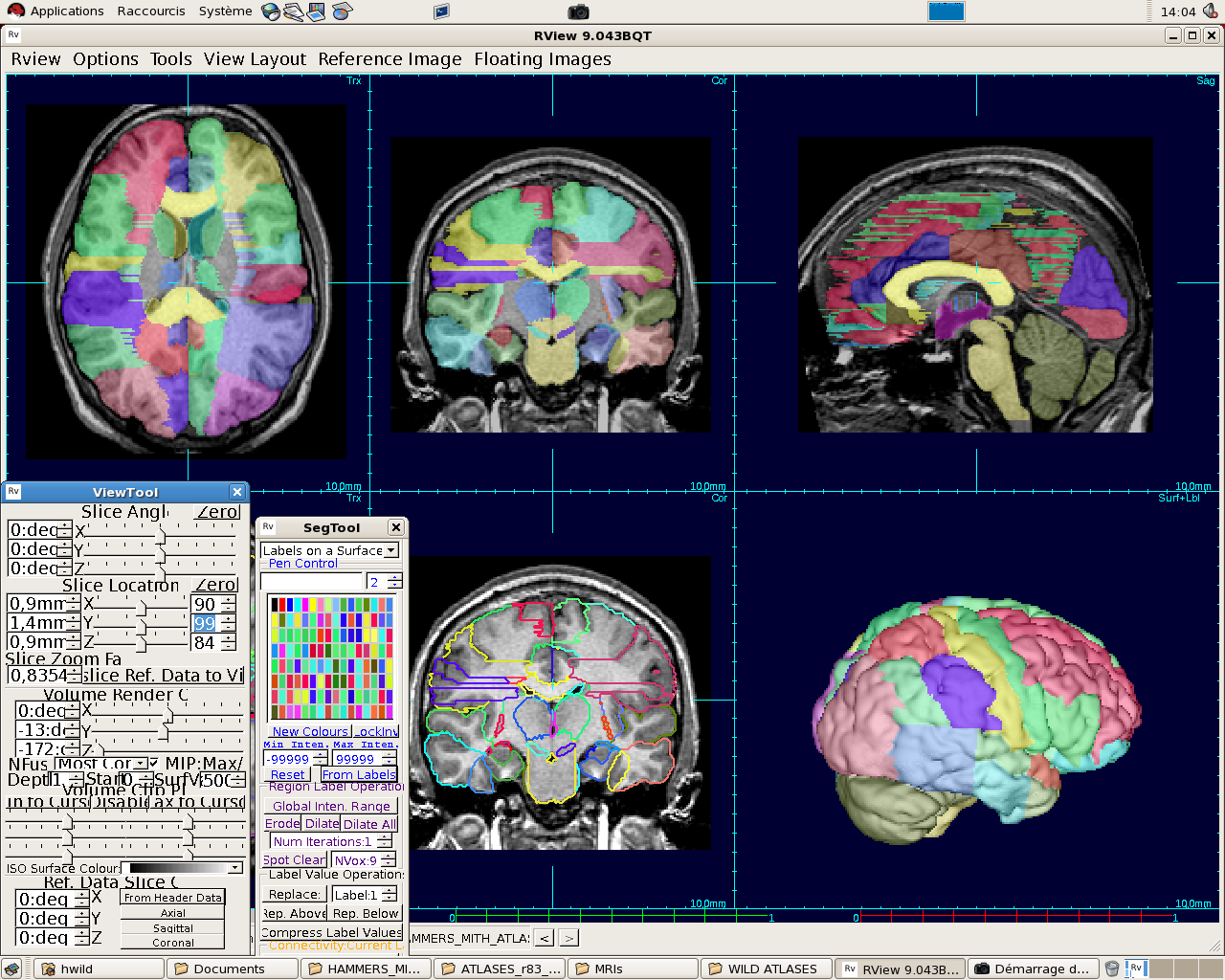


Figure SMG-11. SMG nestled into PCS (a10 RH)

In some cases, the PCS reaches the SF inferiorly (Figure SMG-12), whereas in others it does not (Figure SMG-13).


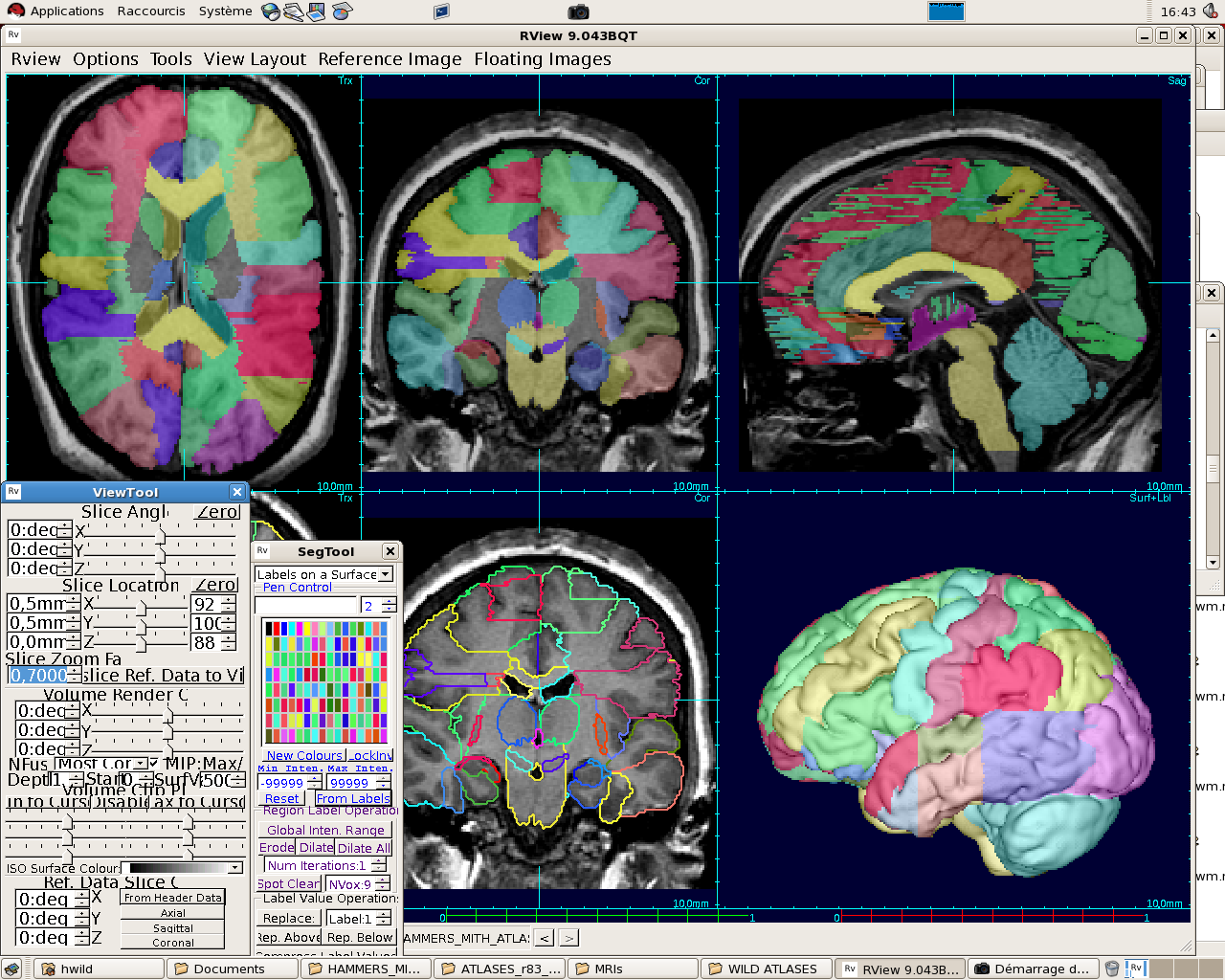


Figure SMG-12. PCS reaches SF inferiorly (a19 LH)


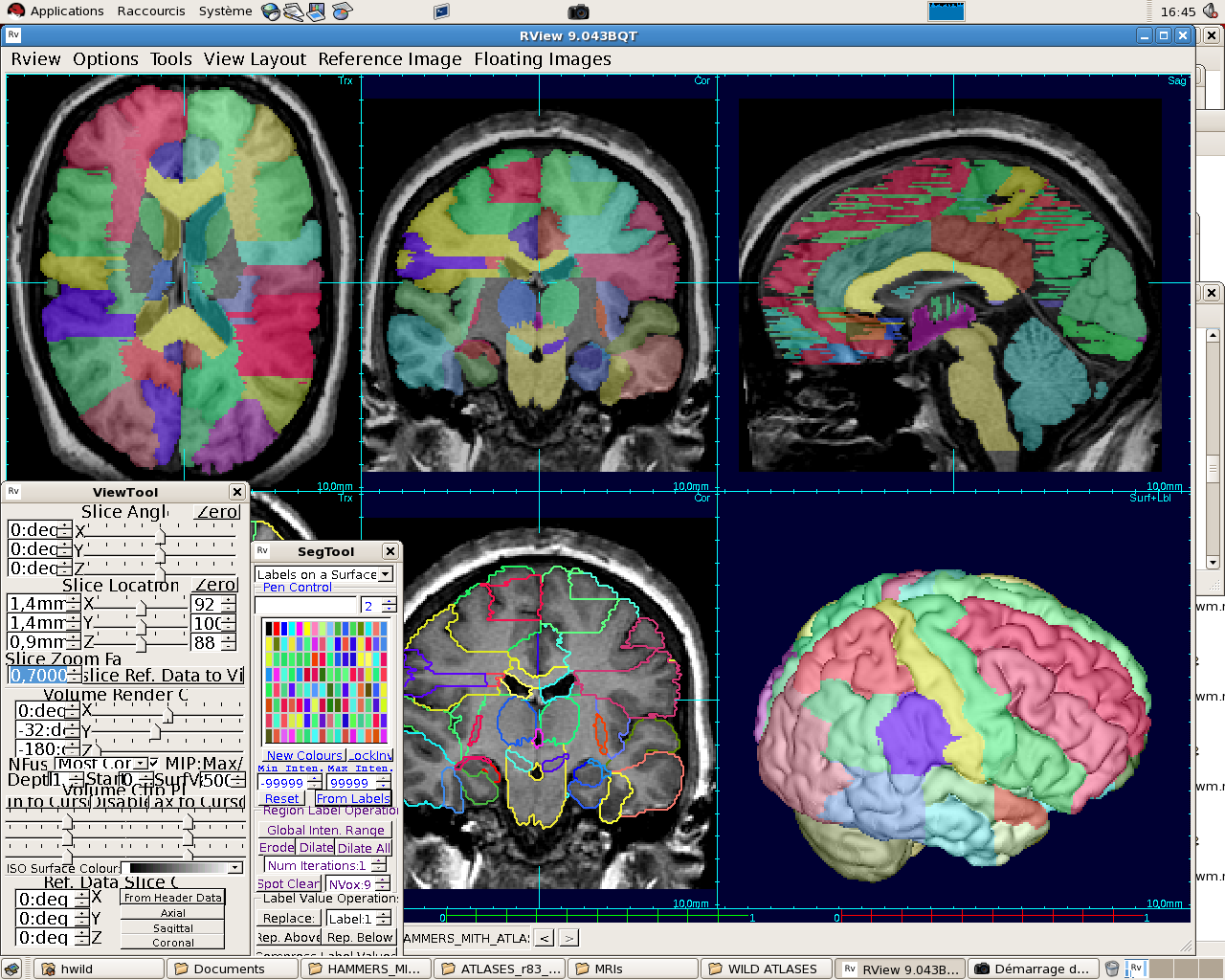


Figure SMG-13. PCS does not reach SF inferiorly (a19 RH)

Care was taken not to overestimate the SMG area by encroaching onto the postcentral gyrus in cases where the PCS does not reach the SF.

**Posterior boundary = PISJ**

This is the most important landmark for the delineation of SMG as it divides the previously segmented intraparietal region, forming a border with the AG. The PISJ is otherwise known as the primary branch of the intraparietal sulcus (IPS). It can be seen by taking several transverse slices. Its presence as a branch from the IPS is recorded. It may form quite complex configurations with neighbouring sulci as shown in Figure SMG-14.

**
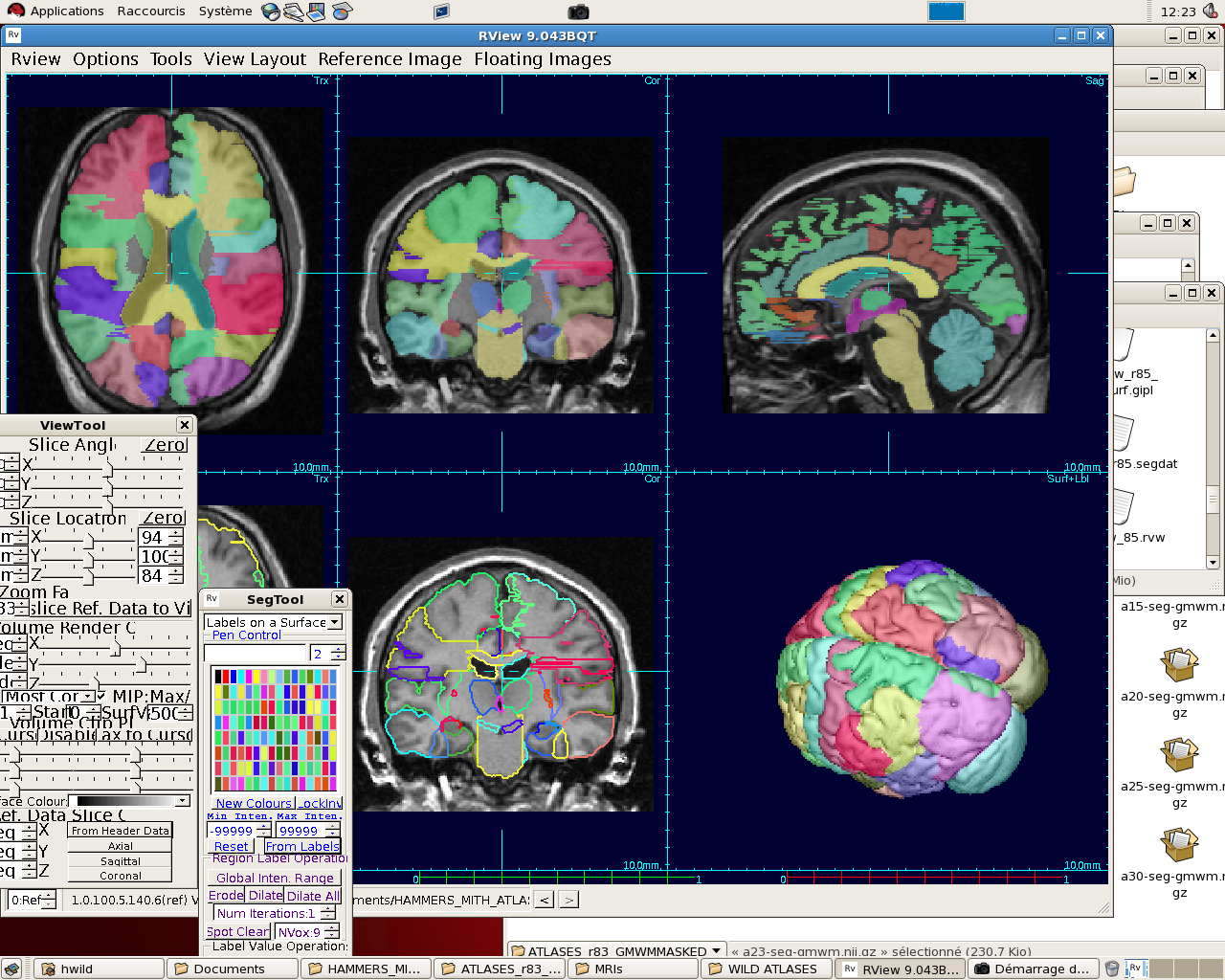
**

Figure SMG-14. Complex configuration of the PISJ (a23 LH)

Ono et al. [5] describe it as being clearly present in 24% of the population in the R and 80% in L hemisphere. In our experience, the PISJ was often prominent (Figure SMG-15).


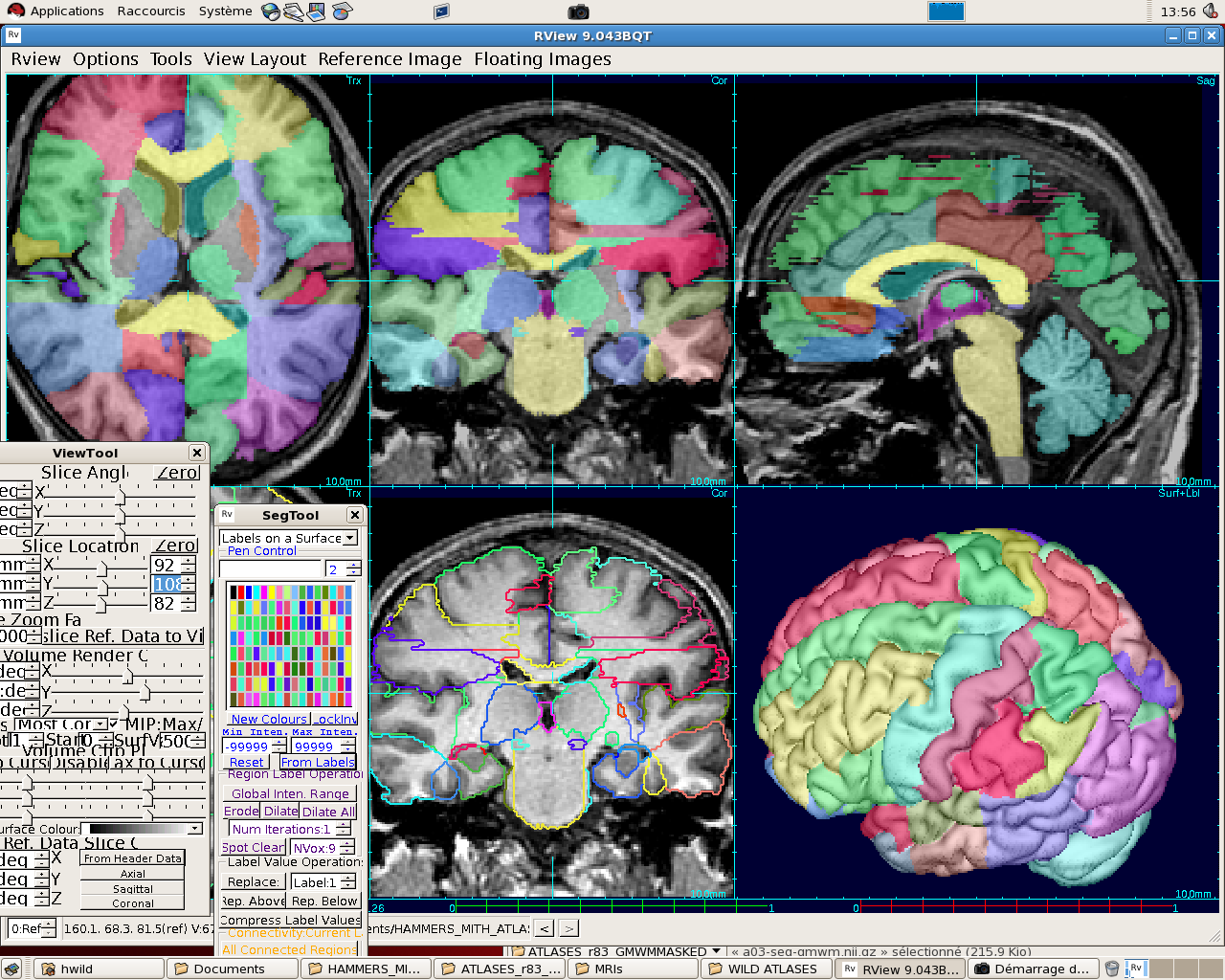


Figure SMG-15. Prominent PISJ (a03 LH)

However, there were also several instances when the sulcus was present as a vaguer indentation albeit in the expected position (Figure SMG-16).


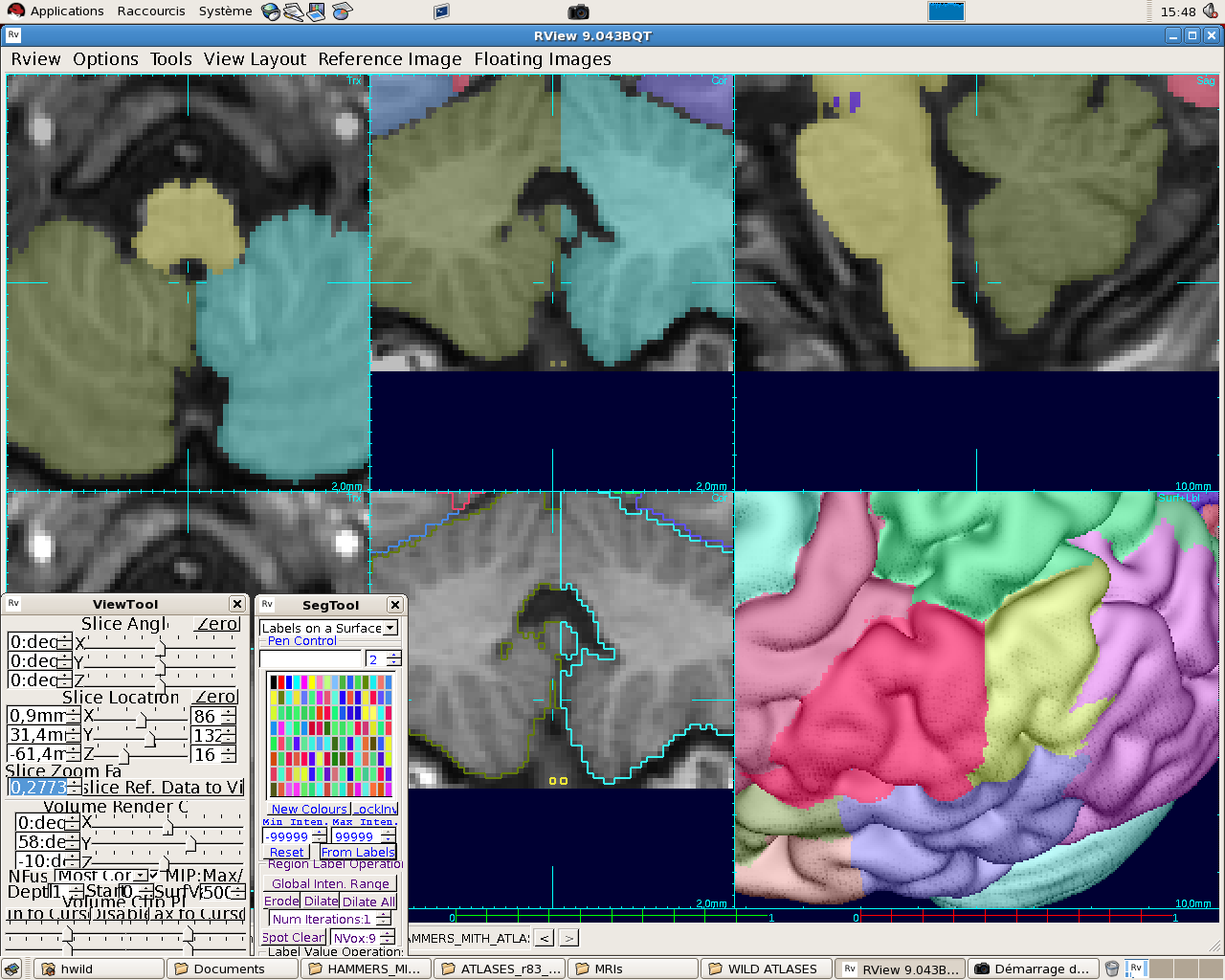


Figure SMG-16. Vague PISJ (a27 LH)

Sometimes only a dimple in the IPS was noted, marking the position of the PISJ (Figure SMG-17).


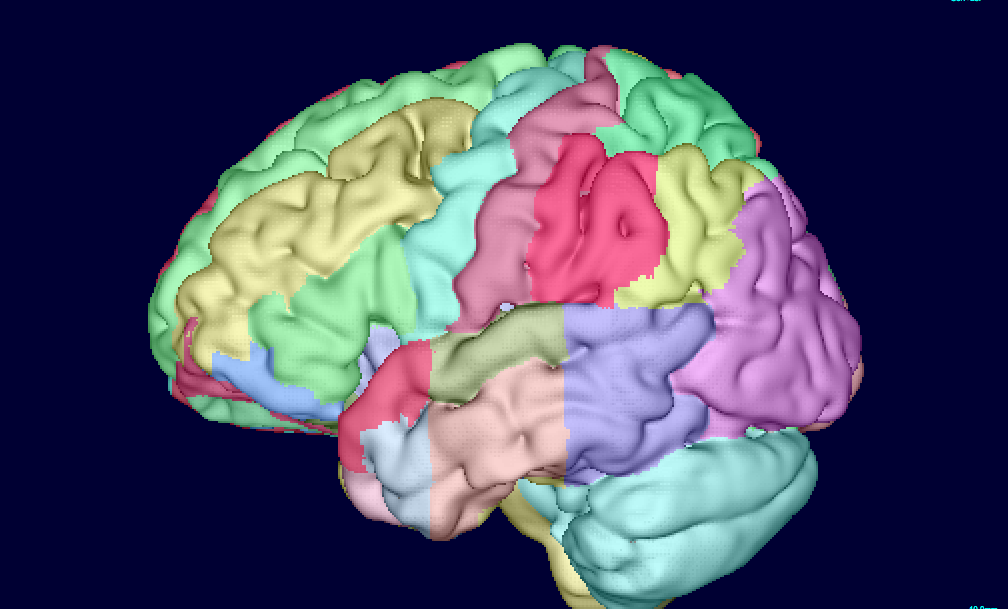


Figure SMG-17. PISJ only present as a dimple (a06 LH)

In two hemispheres a first branch of the IPS was situated anterior to the SF. Declaring this to be the PISJ would have made the SMG unrealistically narrow. In this case the labelling was started at the first sulcus posterior to the SF (Figures SMG-18 and SMG-19).


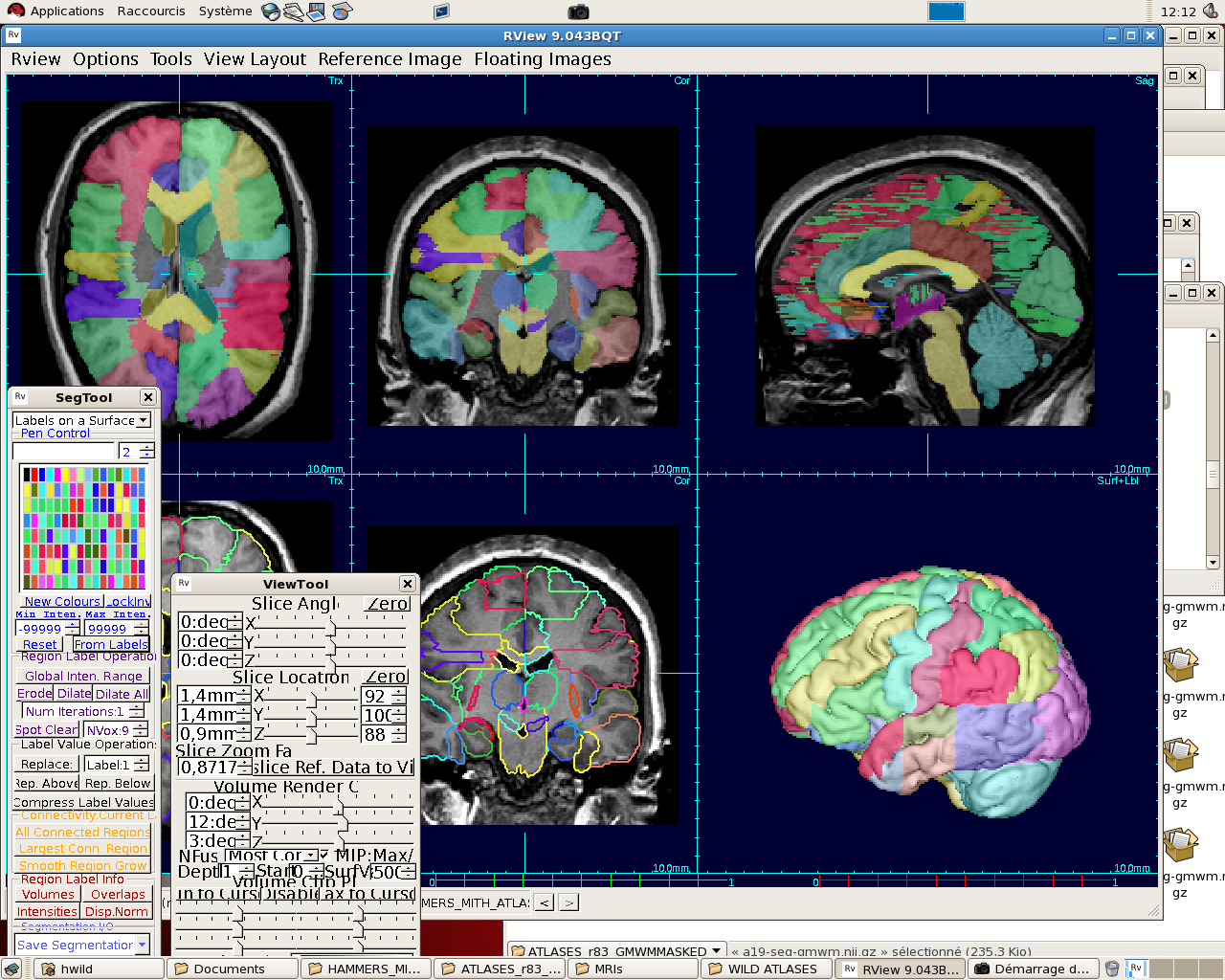


Figure SMG-18. First branch of the IPS anterior to ascending part of the SF (a19 LH).


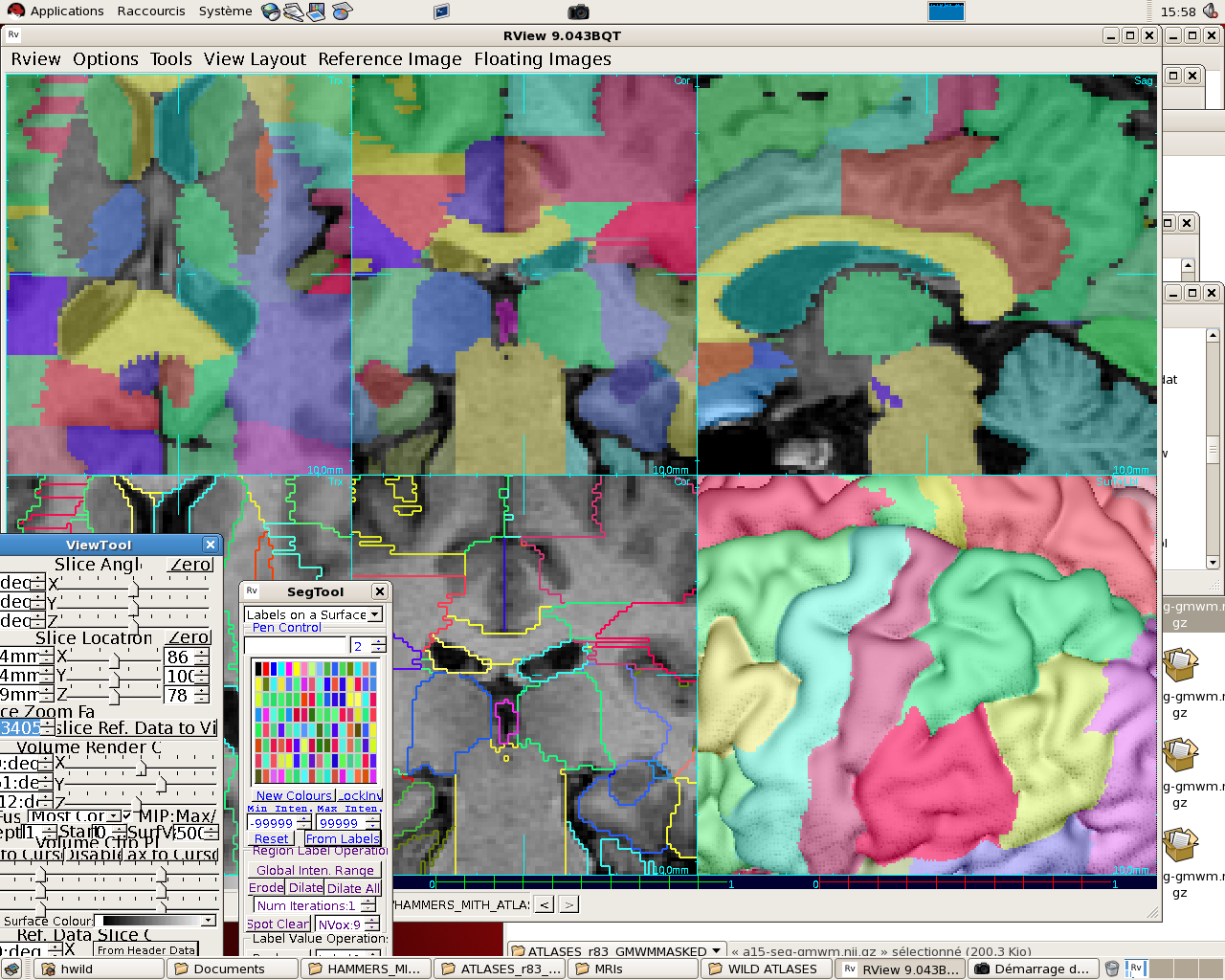


Figure SMG-19. First branch of the IPS anterior to ascending part of the SF (a15 LH).

This enables the SMG to form a horseshoe shape which spans the SF. The labelling follows the PISJ to delineate a continuous posterior border for the SMG. This continuous border line sometimes involves crossing a gyrus or indeed jumping between sulci.

When the PISJ has an anterior to posterior course only the area below the PISJ is labelled. This does leave a triangle of cortex between the IPS and PISJ which is then assigned to the AG.

**Medial and lateral boundaries**

These had been previously defined in the development of the segmented data set [2]. For the division of the intraparietal region into SMG and AG the borders of the SMG were defined primarily from the surface view (Figure SMG-20) and often included additional sulci between the SF and STS (Figure SMG-21)


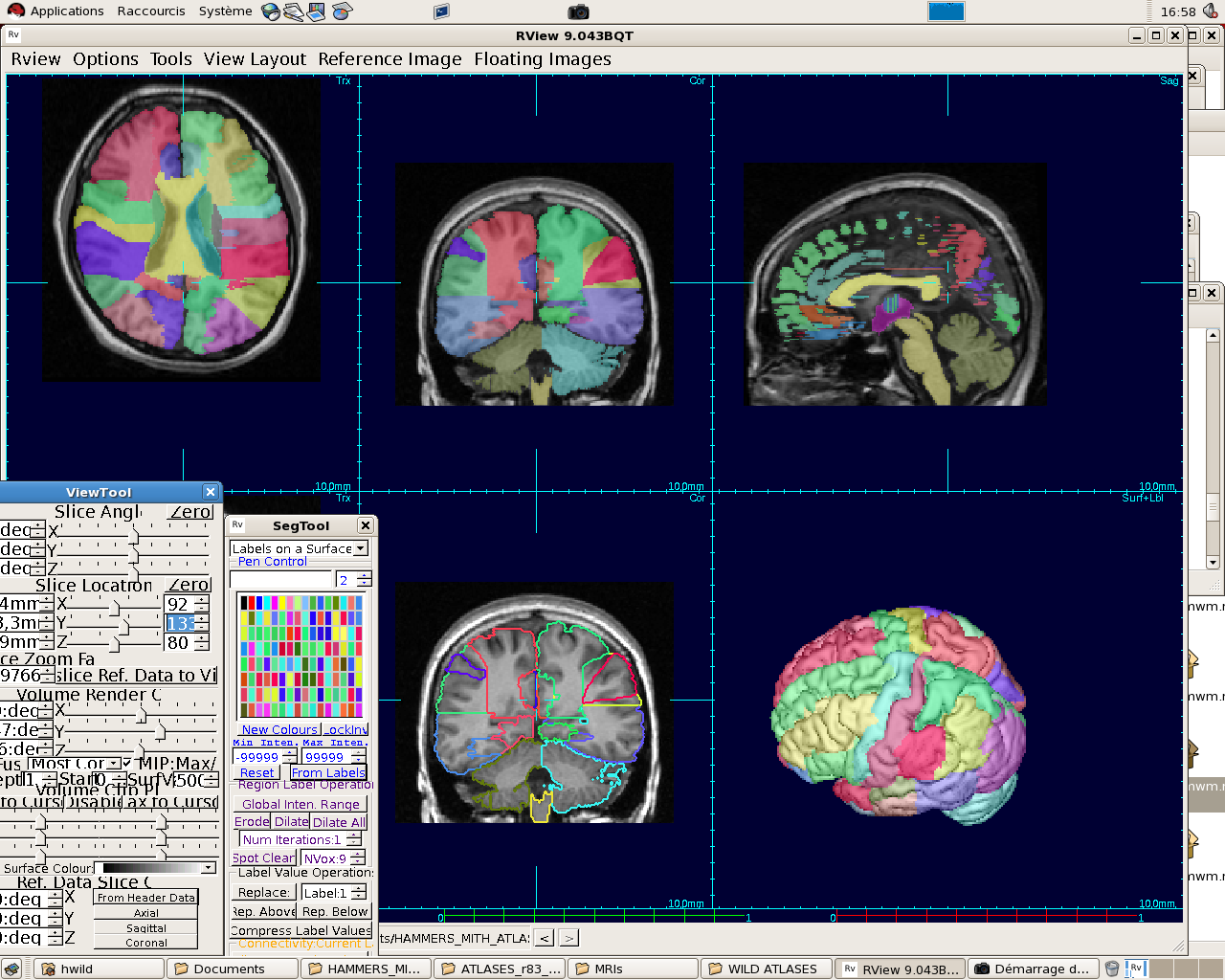


Figure SMG-20. Surface view (a25 LH) showing quality control of boundary definitions.


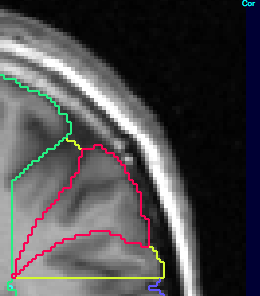

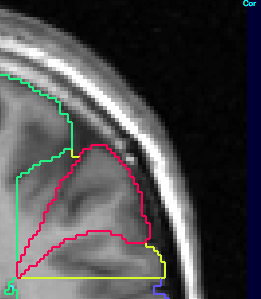


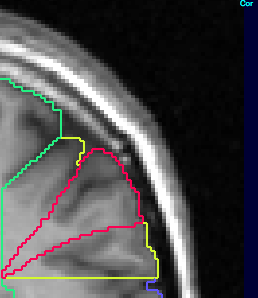


Figure SMG-21. Consecutive medial delineations showing relationship between SMG (red) and AG (yellow) within a single hemisphere (a25 LH). Here, SMG is nestled into a vertically concave anterior boundary of AG, determined largely via surface views (cf. Figure SMG-20).

All WM is assigned to overlying GM, leaving none unaccounted for in future work.

Laterally, all the GM is included for the area. If the space between adjacent sulcal walls is relatively large, the delineating line is taken as the central dividing intrasulcal line. In the case where an island of SMG forms through two sulci fusing in the WM, only the lateral part forming the island is labelled and the labelling is not extended into the more medial separated area (see example in Figure SMG-2, but in contrast to Figure SMG-21, where the boundary is taken to the most inferomedial corner). If the sulcal space is smaller than one voxel (and would therefore be fused in the resulting image), this small space ended up assigned to one or the other side of the sulcal wall.

Care has to be taken in delineating when there are convolutions of the GM**,** especially when one wall passes under another or, when a neighbouring sulcus crosses into the SMG region.

**ANGULAR GYRUS - PROTOCOL**

**Overview**

The angular gyrus (AG) is part of the inferior parietal lobe. It forms a horseshoe shape around the posterior end of the superior temporal sulcus (STS), also known as the Angular Sulcus (AS). The AG lies posterior to the supramarginal gyrus (SMG). It corresponds largely to Brodmann’s area 39 [3] (p. 140 and Figure 85, p. 131) and has been implicated in language functions**.** Anatomically it is separated from the SMG by the primary intermediate sulcus of Jensen (PISJ), which branches from the intraparietal sulcus (IPS). Posteriorly the AG is bordered by the occipital lobe (OL).


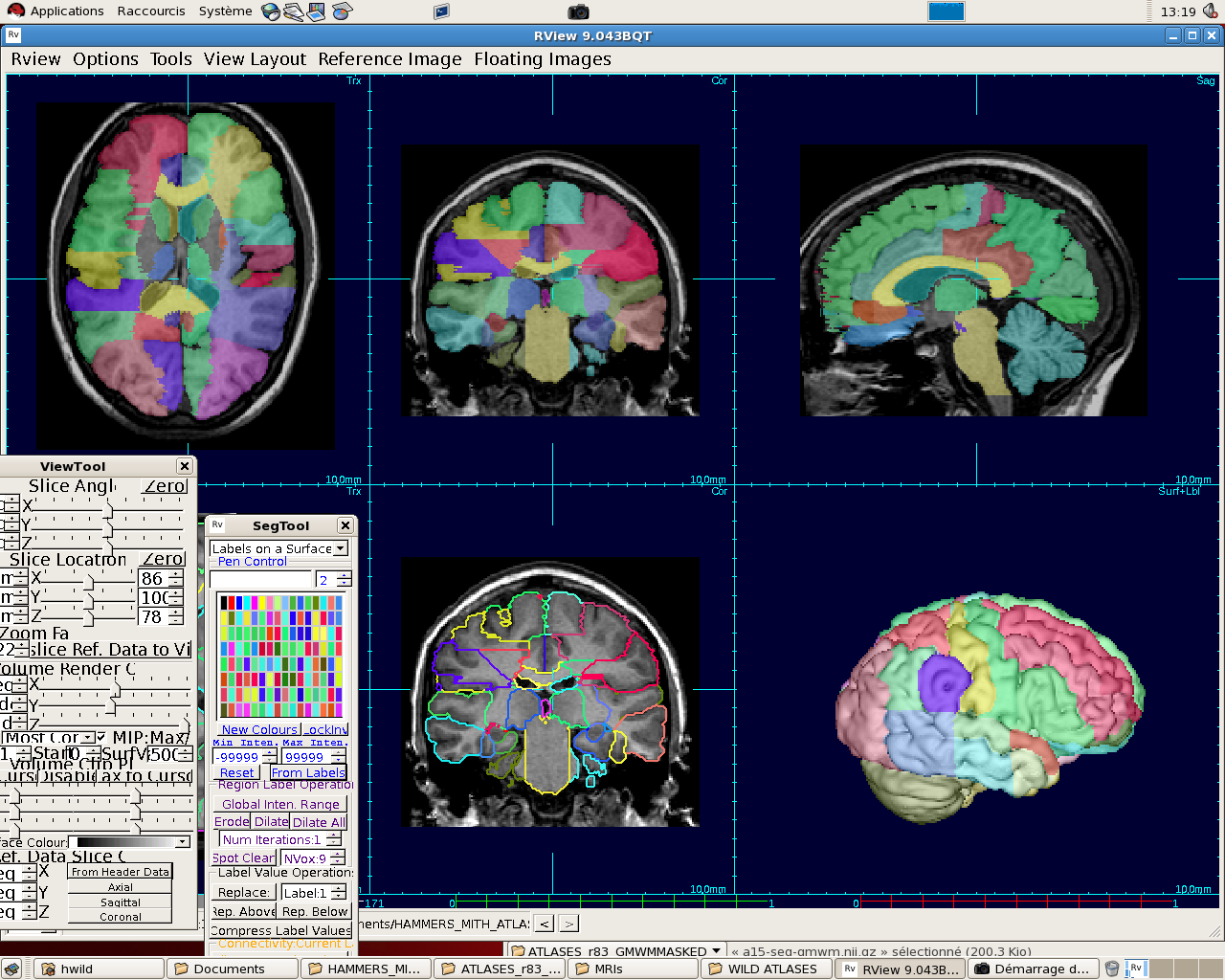

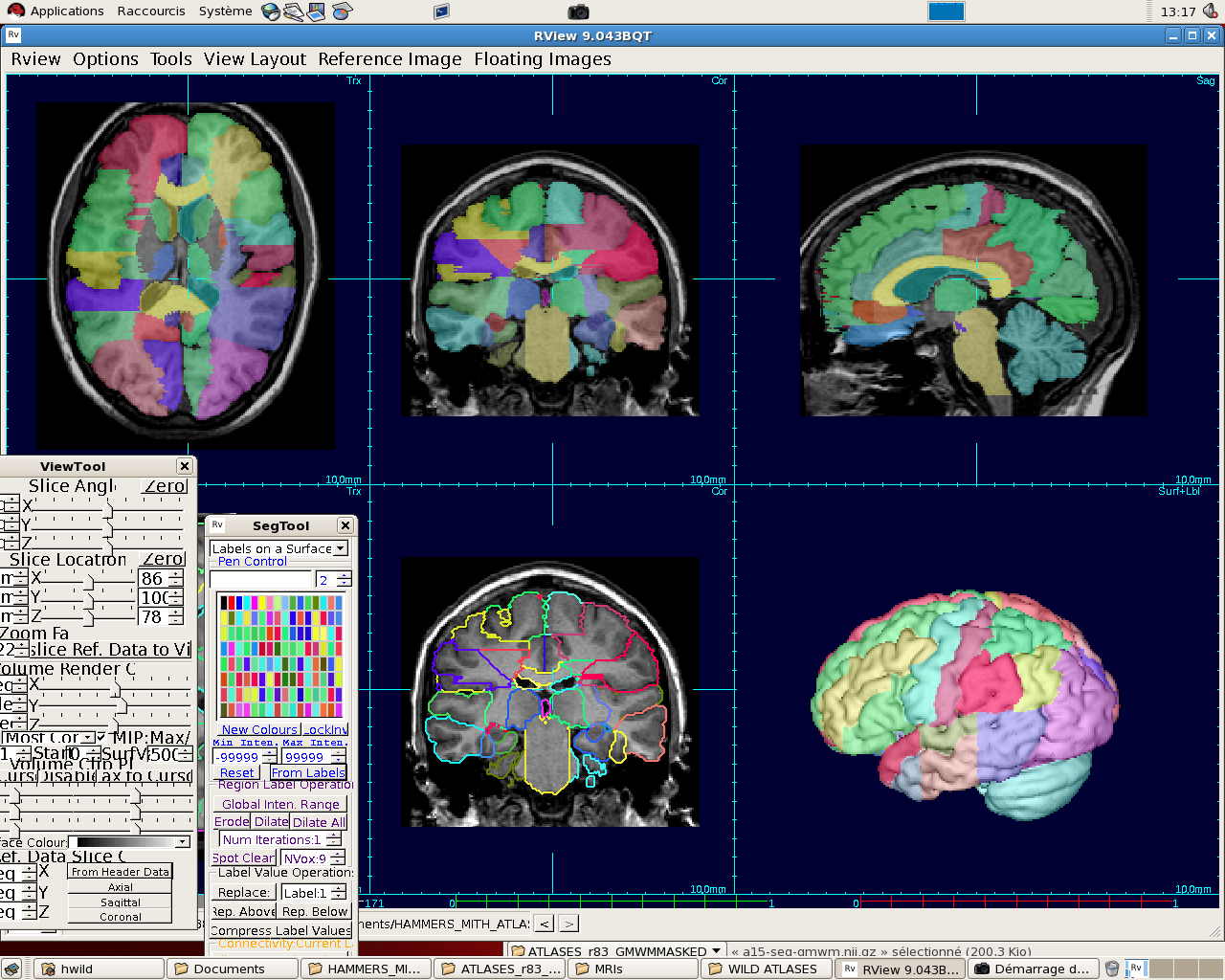
Figure AG-1. Surface views of AG, atlas 15, left and right hemisphere (a15 LH and a15 RH)

The reference boundaries were established using brain atlases, publications, and other protocols (mainly [1,2,4,5] and LONI protocols, http://cms.loni.ucla.edu/ncrr/protocol).

As for the SMG, this study continues the brain atlas work of Hammers et al. [1,2] and includes the areas previously established by their protocols. The parietal lobe had first been labelled as areas 32 and 33 [2] and then later the superior parietal gyrus (SPG) had been delineated as areas 62 and 63 [1]. The SMG protocol above has defined SMG as areas 84 and 85; the remaining parts of areas 32 and 33 form the body of the AG, but are verified and altered where necessary.

We used coronal and surface views to find the deepest point of the STS in a mid-way position and then to follow it anteriorly and posteriorly, in order to decide on its vertical course. The AG curves around the superior end of the STS (angular sulcus).

***Consideration and rejection of an alternative protocol***

In the present study we initially considered a variant (“protocol P*”) of the previously described protocol (“protocol P”) for inferior and medial boundaries for area 32 and 33 [2]. Protocol P* uses the STS as the most inferior border of the AG and thus extends the AG ventrally and medially. Protocol P* would be in accord with the LONI brain protocols.

Ono et al. [5] (p78) describe about 90% of brains as having a clear STS as well as AS, with the AS taken as the ascending posterior branch of the STS. A double ending of the STS towards the angular and anterior occipital sulci was seen in 28% cases for the right hemisphere and 44% for the left hemisphere. The branch of the STS to the OL would form the inferior border of the AG in Protocol P*.

Use of the STS as the inferior border meant that the anterior AG often extended more ventrally than in the protocol used previously. However, it was limited by the inferior boundary described in protocol P posteriorly when no STS was present more ventrally. Once the STS was found, labelling was performed on coronal sections, following the sulcus to its most medial point and then drawing a line vertically to the SMG/AG border. This SMG/AG border took precedence if the STS continued medially inferior to the SMG. When the course of the STS undulated (double-dips) then all the area superior to it was labelled, not just the most lateral part. This continued until the Anterior Occipital Sulcus (AOS), where present, was reached, or the imaginary line taken as the anterior border of OL (see below).

However, due to the variability of the course of the posterior STS, we did not find protocol P* to be consistently applicable. Protocol P* was therefore abandoned, and we remained with the use of protocol P.


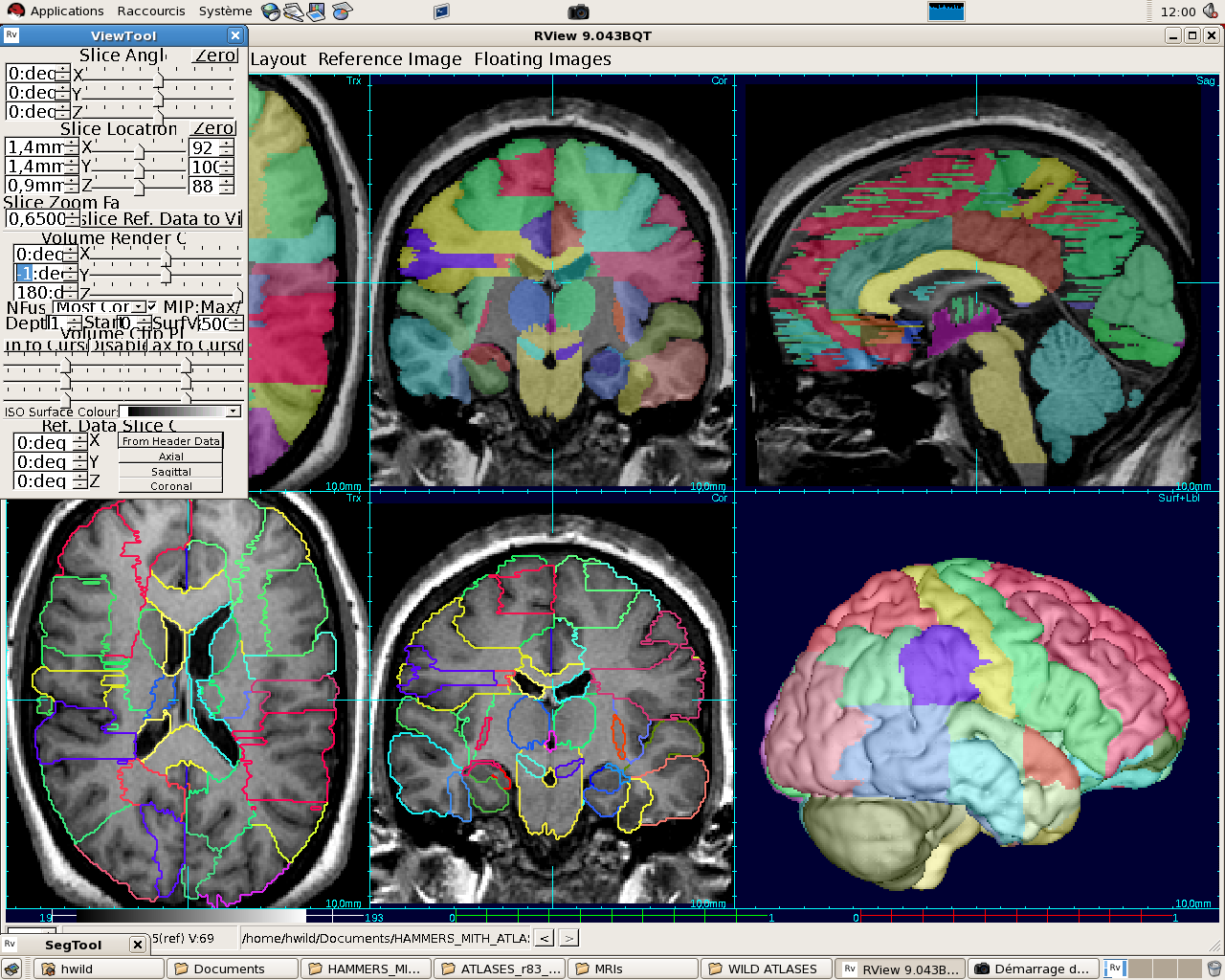


Figure AG-2 (a19 RH). Illustration of difficulties in using STS as the inferior boundary for angular gyrus (interrupted sulcus, unclear anterolateral extension)

**Final protocol**

The AG is delineated according to the following boundaries, using the Rviewqt software.

Coronal views are most often used, with transverse slices where mentioned. Surface views should continuously be available.

**Superior boundary = IPS**

See SMG protocol above and Figure AG-3.


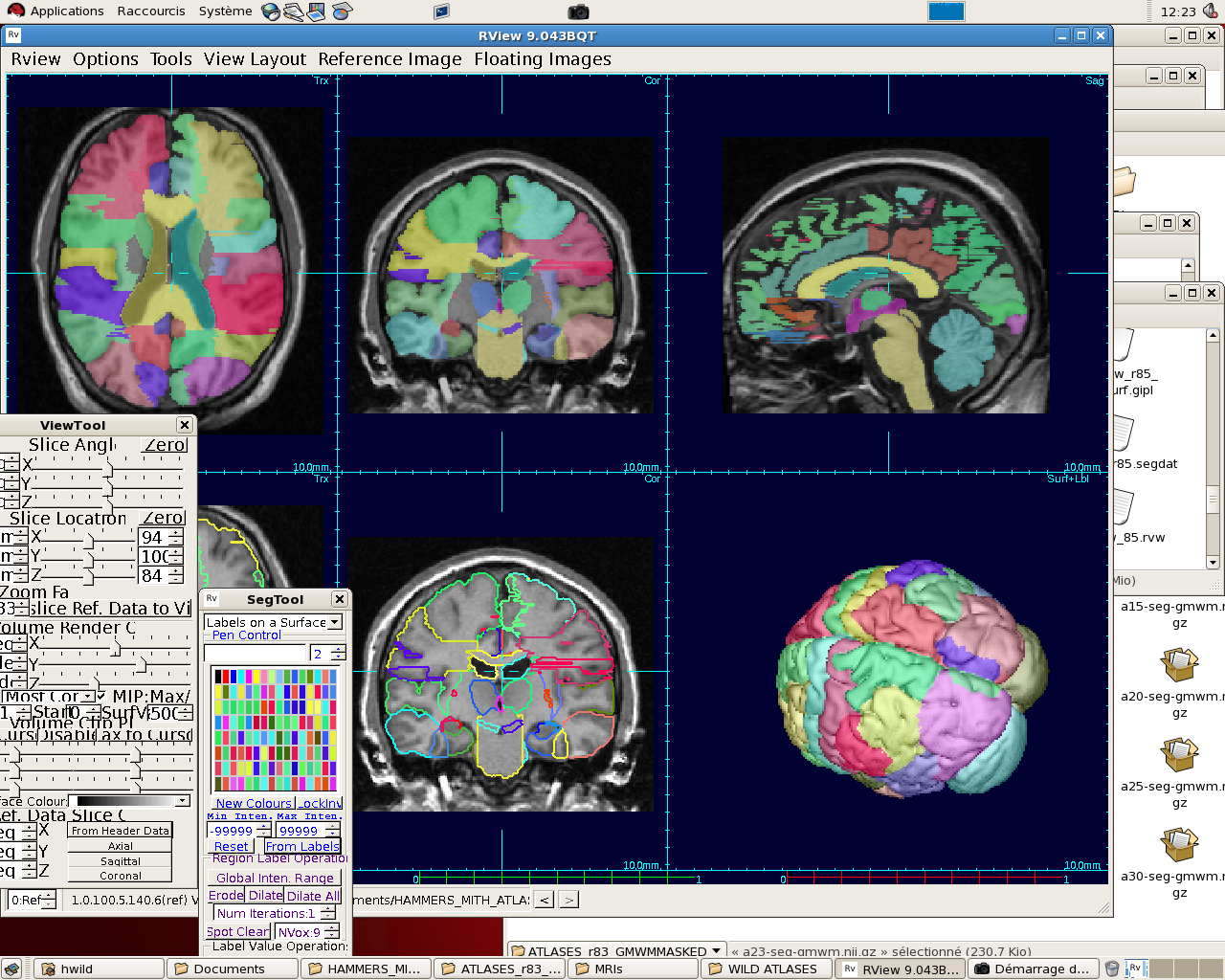


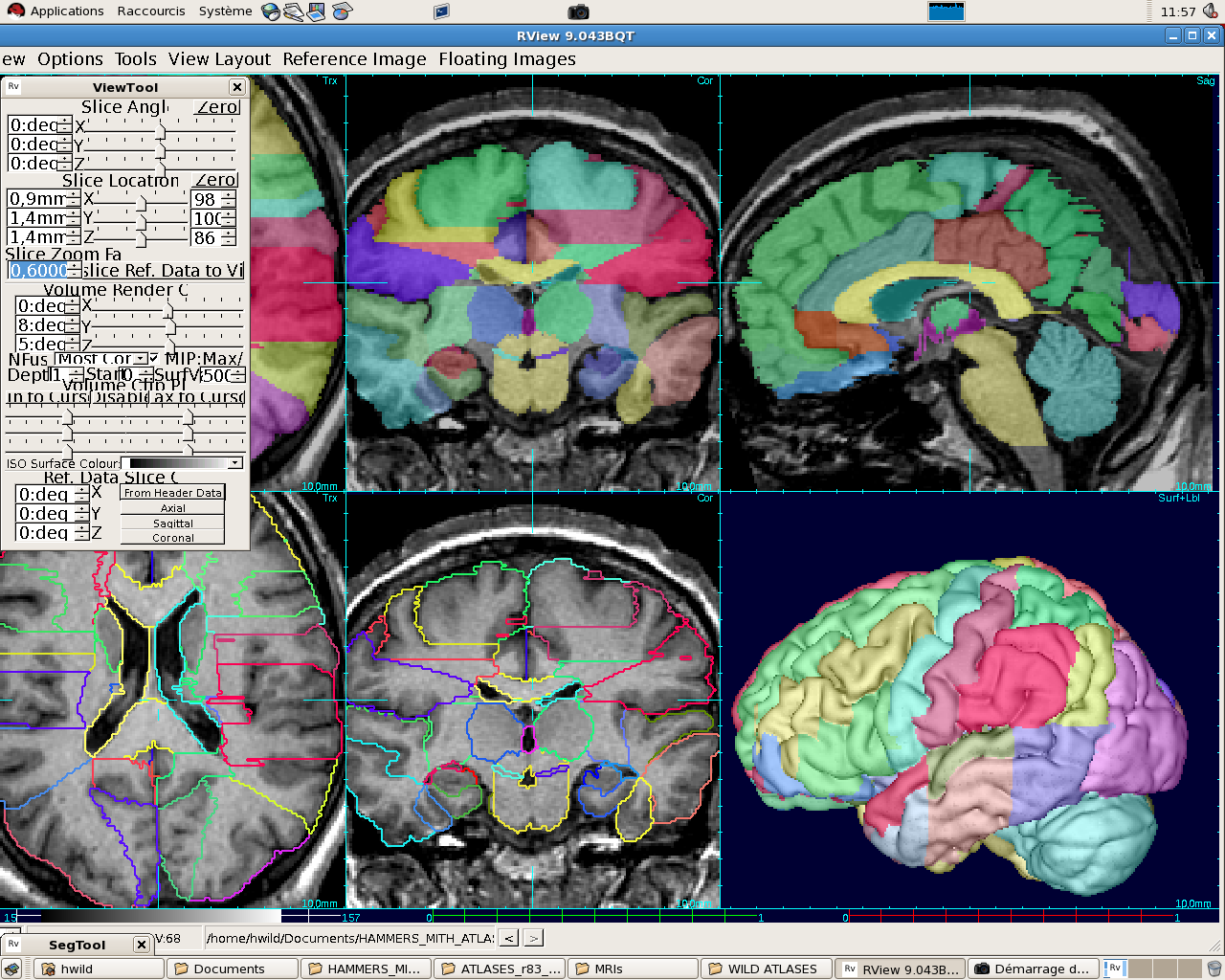


Figure AG-3. Surface view of the IPS. The coronal view shows the IPS to be the deepest sulcus in the area (arrow; see also SMG protocol).

**Inferior boundary**

We use the border previously described as the superior border of areas 30 and 31 [2]. Using transverse views, this is given as the ‘last slice on which the posterior border(s) of any of the temporal lobe structures 9-16 occupied the majority (greater than 50%) of the space between CSF laterally, and non-temporal lobe structures medially’. The inferior boundary of the AG is therefore determined by the overall structure of the mid-to-posterior temporal lobe rather than a single sulcus.

**Anterior boundary = SMG**

The border between the AG and the SMG is found using the PISJ. The presence and appearance of the PISJ is described in the SMG protocol. In delineating the anterior border of the AG, the PISJ is followed and then the border line is continued ventrally until it reaches the inferior boundary. The surface view is crucial in detecting and maintaining the horseshoe shape of both AG and SMG (Figure AG-4).


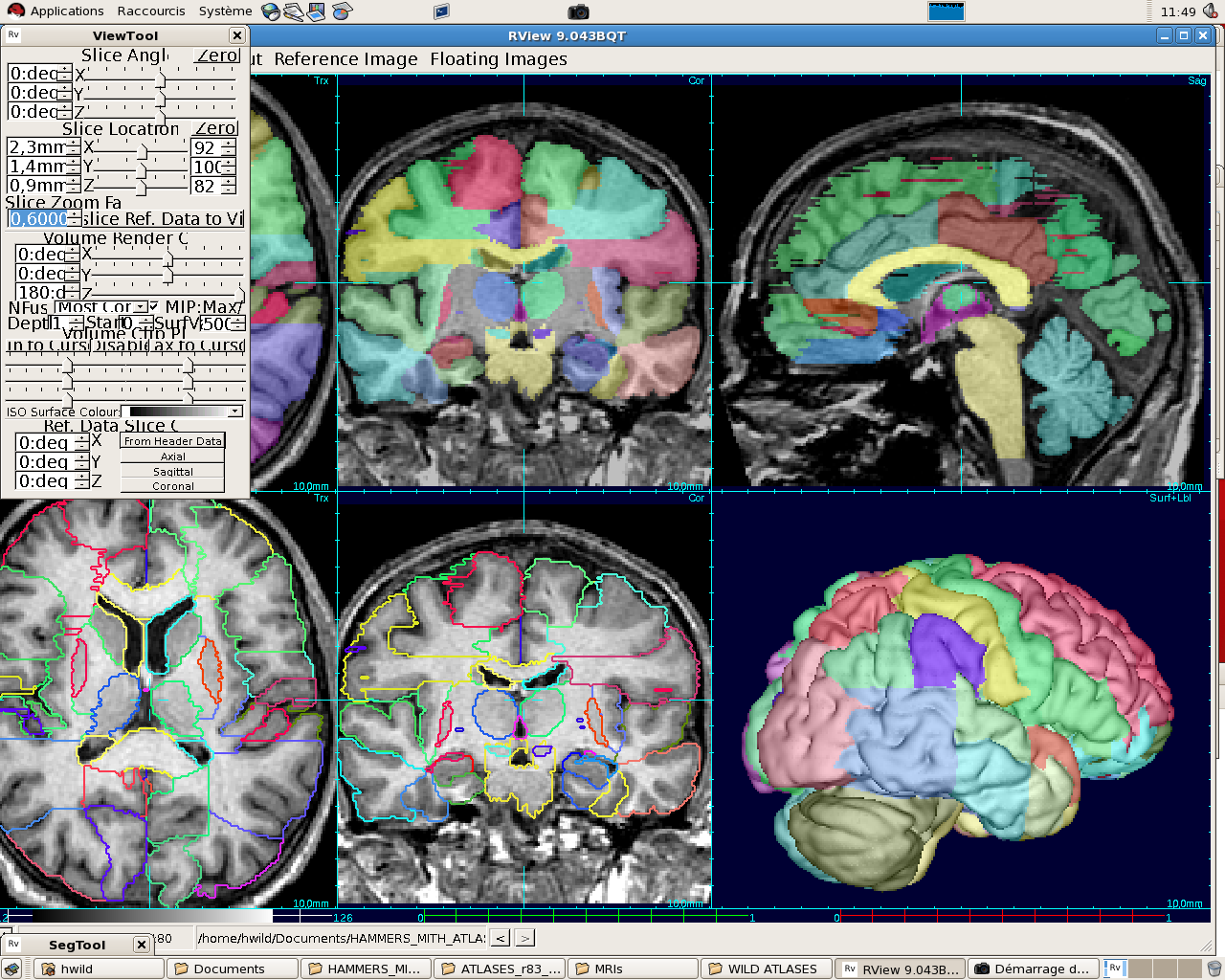


Figure AG-4 (a03 RH). Surface view of AG (in green posterior to SMG)

While it is usually possible to follow sulci, sometimes a gyrus must be crossed (Figure AG-5).


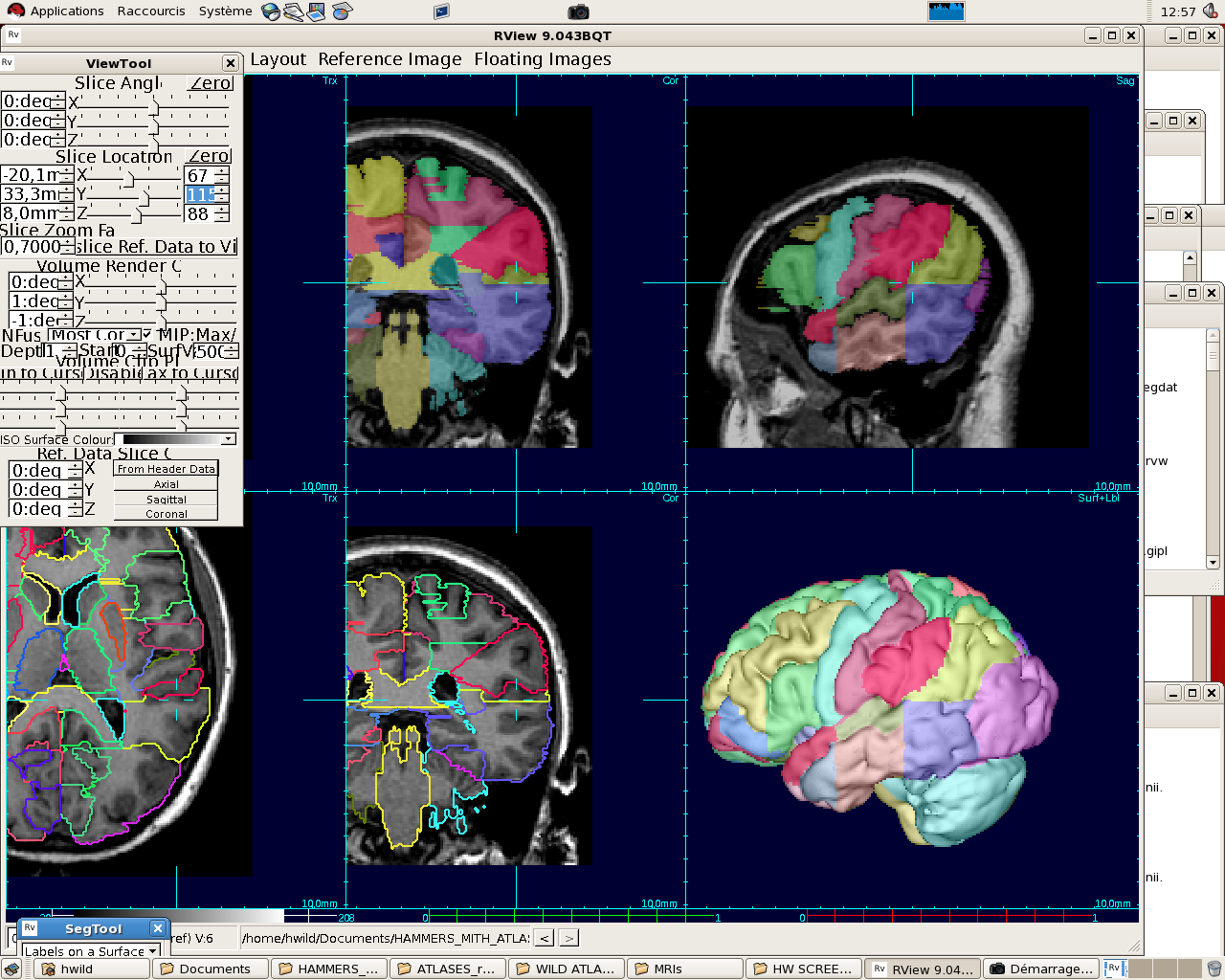


Figure AG-5 (a01 LH). Boundary delineation crossing gyrus.

**Posterior boundary = anterior boundary of OL**

The lateral OL border as operationalized for these protocols corresponds approximately to a line on the convexity from the temporo- occipital notch on the infero-lateral surface to the point where the parieto-occipital fissue cuts into the medial surface (Figure AG-6). The line follows the AOS where this is present, or any sulcus lying on the curved path [2]. It was only occasionally revised to correct small inconsistencies.


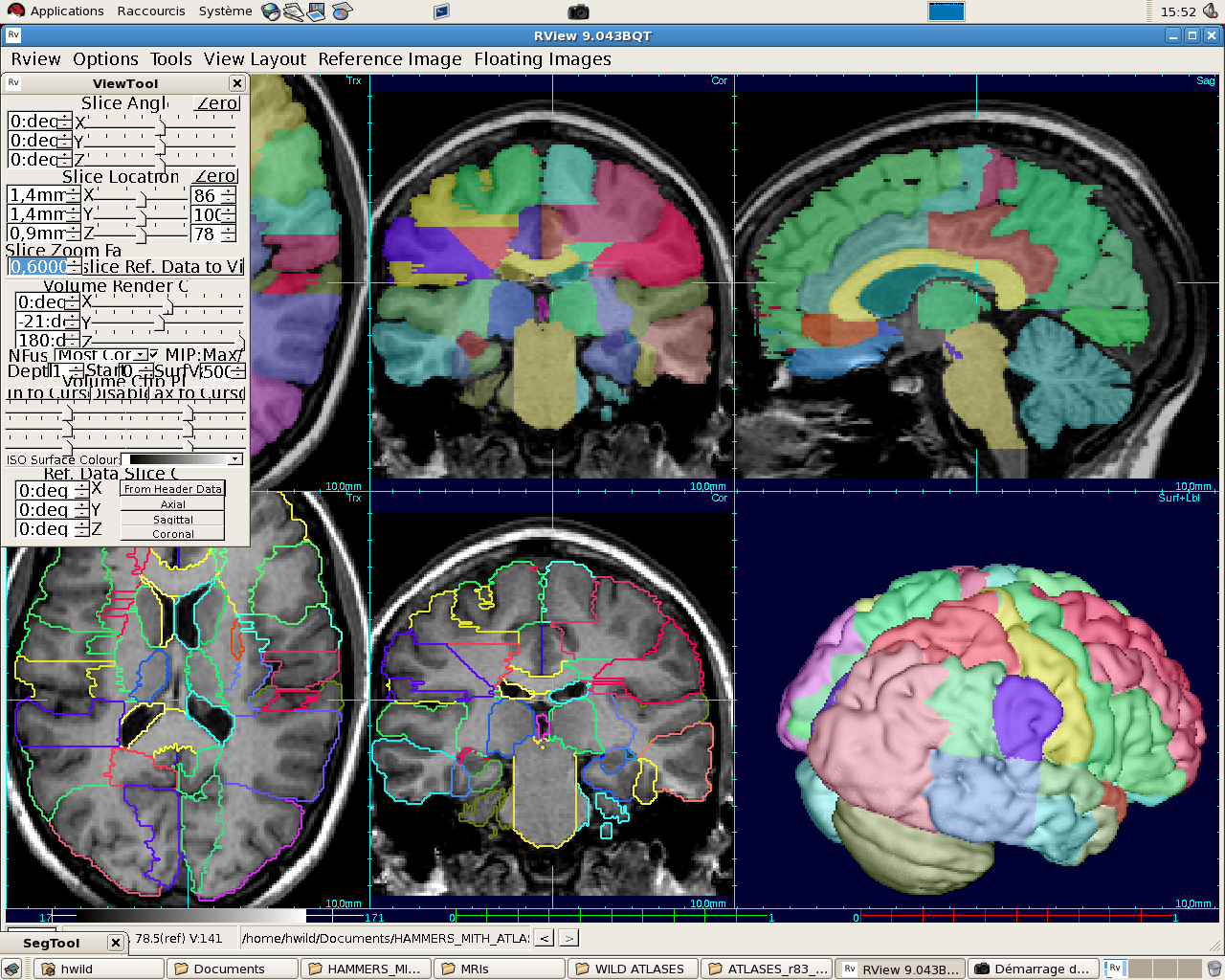


Figure AG-6 (a15 RH). Posterior boundary of AG.

**Medial boundary**

The AG together with the SMG form the inferior parietal area and lie inferolaterally to the superior parietal gyrus (SPG). The SPG label numbers are 62 and 63 [1]. The lateral border of the SPG is the IPS and forms the medial border of the inferior parietal area. On coronal slices, starting posteriorly, a line had been drawn from the deepest end of the IPS initially to the border with the occipital lobe, then more anteriorly to the deepest end of the parieto-occipital fissure, and finally to the deepest end of the anterior calcarine sulcus. Anteriorly, the anterior calcarine sulcus may be included in the posterior temporal lobe structure as previously defined. The line from the deepest end of the IPS had then been continued to the most superior part of the lateral ventricle (cf. [1]).

**Lateral boundary = CSF**

The lateral boundary was revised and refined to match the surface of the brain as closely as possible, and to follow sulci inwards when they had a width of over one voxel.

**Bibliography for Appendix**

1. Gousias IS, Rueckert D, Heckemann RA, Dyet LE, Boardman JP, Edwards AD, et al. Automatic segmentation of brain MRIs of 2-year-olds into 83 regions of interest. NeuroImage. 2008;40: 672–684. doi:10.1016/j.neuroimage.2007.11.034

2. Hammers A, Allom R, Koepp MJ, Free SL, Myers R, Lemieux L, et al. Three-dimensional maximum probability atlas of the human brain, with particular reference to the temporal lobe. Hum Brain Mapp. 2003;19: 224–247. doi:10.1002/hbm.10123

3. Brodmann K. Vergleichende Lokalisationslehre der Großhirnrinde. Leipzig: Barth; 1909.

4. Duvernoy HM. The Human Brain: Surface, Three-dimensional Sectional Anatomy with MRI, and Blood Supply., 2nd ed. New York: Springer; 1999.

5. Ono M, Kubick S, Abernathey C. Atlas of the Cerebral Sulci. Thieme Medical Publishers Ltd.; 1990.
